# Opposite Trap‐State Evolution Pathways Govern Endurance and Retention Failure in Amorphous Chalcogenide Memory

**DOI:** 10.1002/advs.78092

**Published:** 2026-09-29

**Authors:** Byung Jun Lee, Won Hee Jeong, Min Kyu Yang, Gun Hwan Kim

**Affiliations:** ^1^ Department of System Semiconductor Engineering Yonsei University Seoul Republic of Korea; ^2^ Department of Artificial Intelligence Semiconductor Engineering Sahmyook University Seoul Republic of Korea; ^3^ Department of Materials Science and Engineering Yonsei University Seoul Republic of Korea

**Keywords:** chalcogenide, drive‐level capacitance profiling (DLCP), endurance, retention, selector‐only memory (SOM), trap states, V_th_ drift

## Abstract

Selector‐only memory (SOM) is a promising next‐generation memory architecture that integrates both selector and memory functionalities within a single two‐terminal chalcogenide device. Despite its structural simplicity, the reliability of SOM devices remains poorly understood at the mechanistic level. Here, the failure mechanisms governing endurance, retention, and threshold voltage (V_th_) drift of GeSbSe SOM devices are systematically investigated through physical and electrical analyses. Under endurance cycling, time‐of‐flight secondary ion mass spectrometry (ToF‐SIMS) reveals a preferential reduction of Se content, reorganizing the trap environment toward shallower states, confirmed by Poole–Frenkel (P–F), Arrhenius, and drive‐level capacitance profiling (DLCP) analyses. Trap shallowing drives a gradual decrease of both V_th_ states, while heteropolar bond depletion weakens the polarity‐dependent trap response asymmetry, narrowing the memory margin and leading to endurance failure. Under retention and V_th_ drift, x‐ray photoelectron spectroscopy (XPS) and Raman spectroscopy reveal that thermal stimulation drives short‐range structural relaxation toward deeper trap states, directly driving time‐dependent V_th_ rise and asymmetric V_th_ drift. Furthermore, cycling‐induced depletion of Se‐related bonding resources amplifies susceptibility to structural relaxation, establishing a self‐reinforcing degradation pathway that accelerates memory margin loss. These findings establish a unified trap‐based framework linking atomic‐scale structural changes to device‐level reliability degradation.

## Graphical abstract

GeSbSe selector‐only memory devices are investigated through physical and electrical analyses to reveal opposite trap‐state evolution pathways governing reliability degradation. Endurance cycling drives Se migration, transitioning traps from deep to shallow states. In contrast, retention and threshold voltage drift induce structural relaxation, transitioning traps from shallow to deep states. Both modes are unified within a comprehensive trap‐based degradation framework.

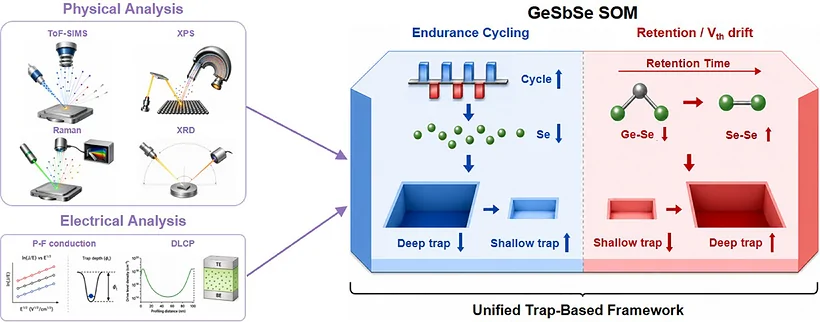

## 1 Introduction

The explosive growth of data‐centric computing driven by artificial intelligence (AI), edge computing, and neuromorphic systems has created an urgent demand for emerging memory technologies capable of bridging the performance gap between dynamic random‐access memory (DRAM) and NAND flash storage. In particular, recent advances in physical neuromorphic computing have demonstrated that the intrinsic nonlinear and dynamic responses of emerging electronic devices can be directly exploited for hardware‐level information processing, further highlighting the importance of advanced device technologies for next‐generation computing systems [1]. Storage‐class memory (SCM) has consequently attracted considerable attention as a promising platform that combines high‐speed operation, non‐volatility, and scalability within high‐density crossbar architectures [2, 3, 4, 5, 6]. In such high‐density crossbar arrays, suppressing unintended current paths through unselected cells is essential for reliable operation, which has motivated extensive development of highly nonlinear selector/access devices. Over the past decade, various selector architectures have been investigated for phase‐change random‐access memory (PCRAM), including p–n junction diodes, ovonic threshold switching (OTS) selectors, and metal–insulator–semiconductor (MIS)‐based structures [3]. P–N heterojunction approaches exploit rectifying transport to suppress leakage through unselected cells, and both PCM‐based heterojunction diodes and hybrid p–n/Schottky diode architectures have been demonstrated as potential access schemes for highly integrated PCRAM [7, 8]. Among these approaches, chalcogenide‐based OTS selectors have been extensively investigated owing to their high drive‐current capability, nanosecond switching speed, low off‐state leakage current, excellent scalability, and high cycling endurance [9, 10, 11, 12, 13]. Across these selector architectures, key parameters including selectivity, off‐state leakage current, drive current, operating voltage, endurance, scalability, and integration compatibility provide important benchmarks for evaluating their suitability for high‐density memory arrays.

Despite substantial progress in selector technologies, conventional crosspoint memory architectures generally require the selector and memory elements to be integrated as separate functional components, resulting in increased structural and process complexity. Against this background, selector‐only memory (SOM) has recently emerged as a next‐generation memory concept that integrates both memory and selector functionality within a single two‐terminal chalcogenide device [14, 15, 16, 17, 18]. As illustrated in Figure 1a,b, unlike conventional one‐selector‐one‐memory architectures that require separate selector and memory elements, SOM achieves a substantially simplified device structure while maintaining polarity‐dependent, non‐volatile threshold voltage (V_th_) modulation behavior.

**FIGURE 1:**
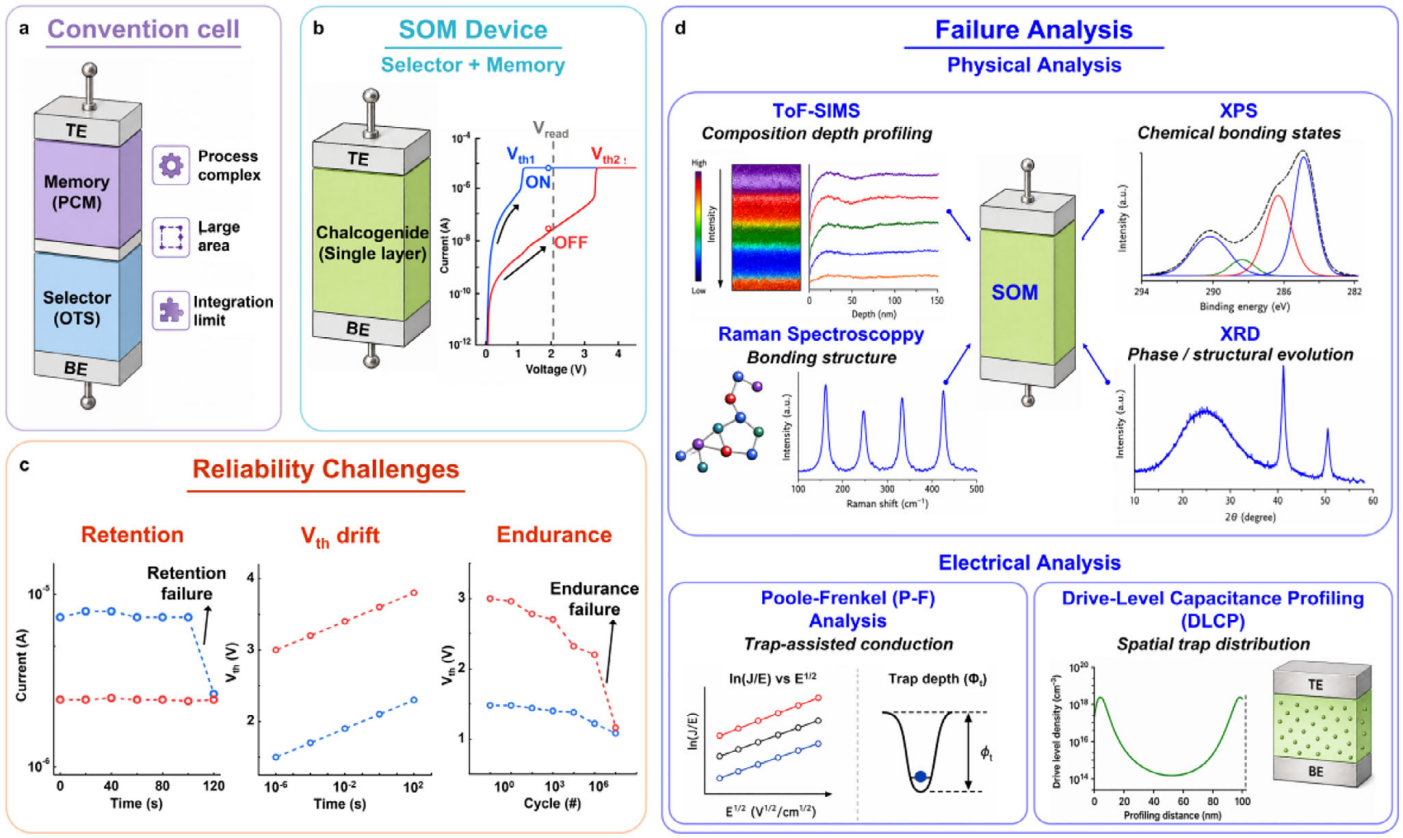
Schematic overview of the SOM device concept, key reliability challenges, and the failure analysis framework employed in this study. (a) Schematic illustration of a conventional one‐selector‐one‐memory (1S1R) cell, highlighting the structural complexity arising from the separate memory (PCM) and selector (OTS) elements, along with the associated challenges of process complexity, large cell area, and integration limits. (b) Schematic of the SOM device structure and representative *I–V* characteristics, demonstrating polarity‐dependent non‐volatile V_th_ modulation (V_th1_ and V_th2_) achieved within a single two‐terminal chalcogenide layer. (c) Schematic representation of the three major reliability challenges in SOM devices: retention failure, in which the read current degrades over time; V_th_ drift, in which V_th_ gradually increases following a write operation; and endurance failure, in which V_th1_ and V_th2_ converge after repeated cycling. (d) Overview of the reliability failure analysis framework adopted in this study, comprising physical analysis (ToF‐SIMS for compositional depth profiling, XPS for chemical bonding states, Raman spectroscopy for bonding structure, and XRD for phase and structural evolution) and electrical analysis (P–F analysis for trap‐assisted conduction and DLCP for spatial trap distribution).

SOM operates through polarity‐dependent modulation of the V_th_ within chalcogenide‐based materials. Depending on the applied programming polarity, the device can reversibly transition between low‐V_th_ and high‐V_th_ states while preserving its amorphous structure, thereby enabling memory functionality without the crystallization processes required in conventional phase‐change memory systems. The difference between these V_th_ states defines the operational memory window for read operation. Although the detailed switching mechanism of SOM devices remains under debate, charge transport in the subthreshold regime is widely described by the Poole‐Frenkel (P‐F) conduction mechanism, suggesting that defect‐mediated trap states play a critical role in determining switching characteristics and memory behavior [11, 19, 20, 21]. In particular, the evolution of trap states under electrical stress is believed to be closely associated with reliability degradation in SOM devices.

For practical deployment of SOM devices as SCM, reliable device operation is essential. As shown in Figure 1c, among the various reliability metrics, endurance and V_th_ drift represent the two most critical challenges. Endurance determines the cycling stability of the device under repeated programming operation, whereas V_th_ drift, analogous to retention degradation in conventional memory devices, describes the gradual shift of V_th_ over time following switching events. These reliability issues directly limit operational read margins and long‐term device stability, thereby hindering practical implementation of SOM technologies [14, 22, 23, 24]. To address these challenges, extensive efforts have been devoted to improving SOM reliability through elemental doping [22, 25, 26, 27, 28], compositional optimization [29, 30, 31], and pulse engineering strategies [32, 33].

However, despite these advances, the fundamental origins of reliability failure in SOM devices remain poorly understood, as most studies have primarily focused on empirical optimization without establishing a mechanistic basis for the observed improvements. In particular, the relationship between electrical degradation behavior and the accompanying structural and compositional evolution within the chalcogenide network has not yet been systematically clarified. Without a mechanistic understanding of how endurance degradation and V_th_ drift emerge during device operation, optimization strategies risk addressing phenomenological symptoms rather than the root causes of failure. Establishing a mechanistic framework that directly correlates trap‐state evolution with material‐level structural changes is therefore essential for the rational design of intrinsically reliable SOM devices.

As summarized in Figure 1d, we present a comprehensive investigation into the reliability failure mechanisms of Ge‐Sb‐Se‐based SOM devices from both electrical and materials perspectives. Electrically, we analyze the evolution of trap characteristics during endurance degradation and V_th_ drift using P‐F conduction analysis and drive‐level capacitance profiling (DLCP), enabling investigation of trap‐depth evolution and trap‐state distributions under reliability stress. We further employ complementary materials characterization—including x‐ray photoelectron spectroscopy (XPS), time‐of‐flight secondary ion mass spectrometry (ToF‐SIMS), x‐ray diffraction (XRD), and Raman spectroscopy—to probe compositional redistribution, bonding evolution, and structural relaxation within the chalcogenide thin films. By directly correlating electrical trap evolution with material‐level structural changes, this work establishes a unified mechanistic picture of reliability degradation in SOM devices and provides fundamental guidelines for the development of high‐endurance and drift‐suppressed SOM technologies.

## 2 Results and Discussion

### 2.1 Structural and Chemical Characterization of GeSbSe Devices

To evaluate the structural integrity and chemical environment of the W/GeSbSe/W device, a combination of high‐resolution transmission electron microscopy (HR‐TEM), energy‐dispersive spectroscopy (EDS), Rutherford backscattering spectroscopy (RBS), and XPS analyses was performed. The active area (A) of the device used in this study is 10 µm × 10 µm.

Figure 2a presents a cross‐sectional HR‐TEM image of the W/GeSbSe/W stack, in which a uniform active layer is observed between the W top electrode (TE) and bottom electrode (BE). The thickness of the GeSbSe switching layer is measured to be approximately 60 nm, and no observable voids or delamination were detected at the electrode/film interface under the magnification and resolution conditions of the present TEM analysis. To further confirm the structural state of the film, Fast Fourier Transform (FFT) analysis was performed on the HR‐TEM image, as shown in the inset of Figure 2a. The GeSbSe layer exhibits a diffuse amorphous halo without crystalline diffraction spots, confirming that the film was formed in an amorphous state. EDS mapping verifies the uniform elemental distribution of W, Ge, Se, and Sb across the active layer, indicating that the co‐sputtering process was successfully carried out and that Sb was effectively incorporated into the Ge─Se matrix without compositional segregation.

**FIGURE 2:**
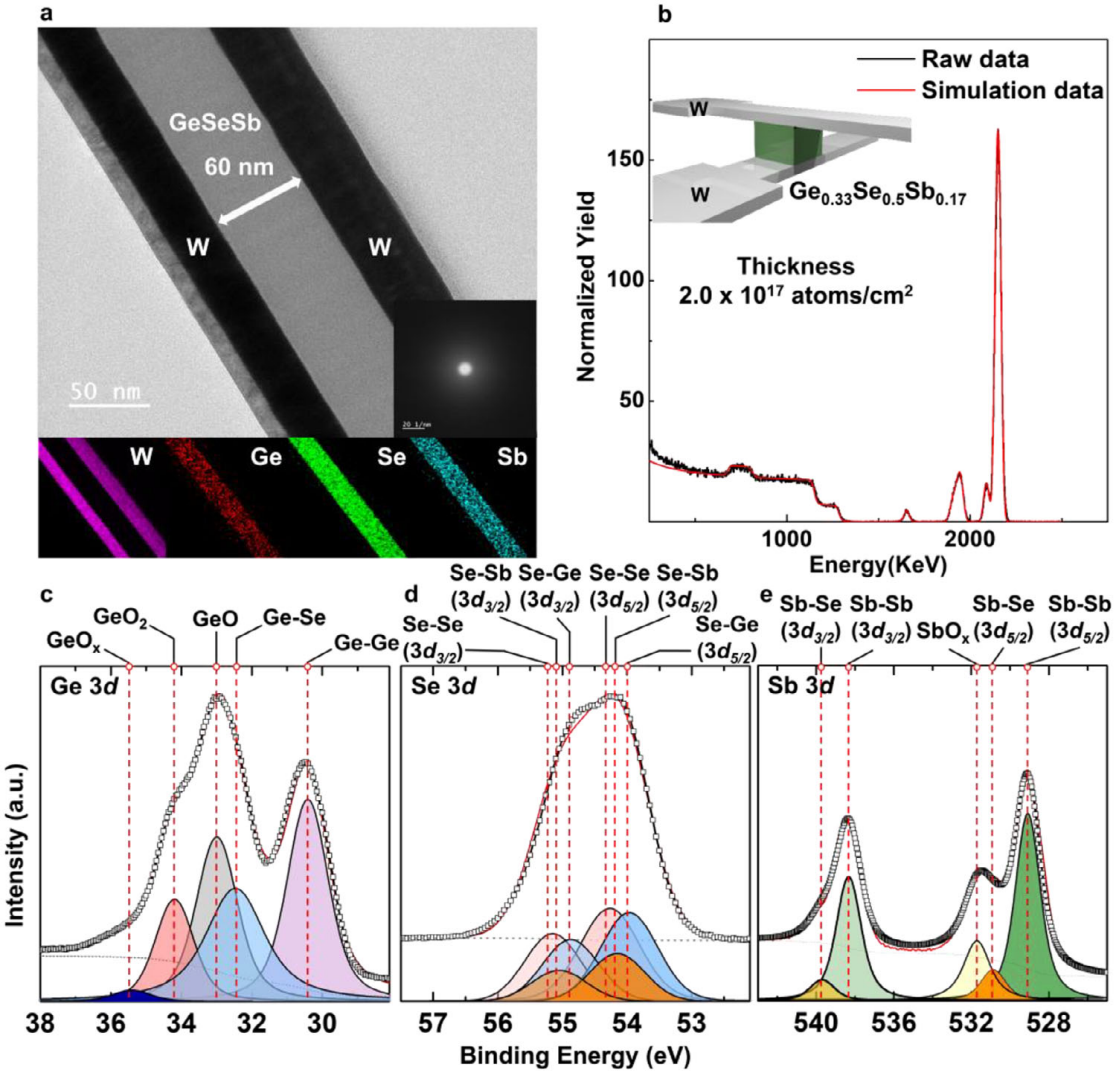
Structural and chemical characterization of the GeSbSe SOM device. (a) Cross‐sectional HR‐TEM image of the W/GeSbSe/W stack, with the corresponding FFT pattern shown in the inset and EDS elemental maps showing the spatial distributions of W, Ge, Se, and Sb across the active layer. (b) Schematic illustration of the device structure and the corresponding RBS spectrum with a simulation curve. (c) Ge 3*d* XPS core‐level spectrum with deconvoluted peak components. (d) Se 3*d* XPS core‐level spectrum with deconvoluted peak components. (e) Sb 3*d* XPS core‐level spectrum with deconvoluted peak components.

To quantitatively assess the chemical composition of the switching layer, RBS measurements were carried out, as shown in Figure 2b. The raw data and simulation curves are labeled to distinguish the measured and fitted scattering profiles, respectively. The fitting analysis confirms a stoichiometry of Ge_0_._33_Se_0_._5_Sb_0_._17_, indicating that Sb is effectively incorporated into the Ge─Se network at a controlled compositional ratio. In Figure 2b, the RBS spectrum shows partially overlapped peaks owing to the similar atomic numbers and masses of Ge, Se, and Sb, where the energy separation between their backscattered ions approaches the detector resolution limit and causes their contributions to merge into broadened features. The Ge, Se, and Sb compositions were therefore obtained by deconvoluting these overlapped features through RBS simulation. These compositional results quantitatively support that the Ge, Se, and Sb elements are uniformly distributed within the GeSbSe film at the intended ratio and provide a compositional basis for interpreting the bonding configurations and trap formation behavior to be discussed in the subsequent XPS and Raman analyses.

To cross‐validate this result, the composition was independently examined by EDS on the cross‐sectional device stack. The atomic percentages of the GeSbSe switching layer determined by RBS and EDS are summarized in Table S1. Renormalizing the measured atomic fractions to the three chalcogenide‐forming elements yields Ge 38.1, Sb 14.7, and Se 47.2 at.%, in reasonable agreement with the RBS values. The slightly higher Ge and lower Se content obtained by EDS is attributed to the L‐series quantification of Se and Sb in the low‐energy region dominated by the W M lines, together with the partial electron‐beam‐induced loss of volatile Se; RBS is therefore adopted as the reference composition throughout this work. Notably, the measured stoichiometry corresponds to a mean coordination number of ⟨*r*⟩ = 2.83, well above the rigidity percolation threshold of 2.4, indicating an over‐constrained network that retains considerable internal structural strain [34, 35]. Moreover, a chemically ordered network in which every Ge and Sb atom is fully coordinated by Se would require approximately 65 at.% Se at the present Ge:Sb ratio, so the film is Se‐deficient by ∼ 15 at.% relative to this limit. This deficiency necessarily implies that a substantial fraction of Ge and Sb atoms must form homopolar Ge─Ge and Sb─Sb bonds, anticipating the bonding configurations revealed by the XPS analysis below.

To investigate the chemical bonding configurations within the GeSbSe layer, XPS analysis was conducted, as summarized in Figure 2c–e. The Ge 3*d*, Se 3*d*, and Sb 3*d* core‐level spectra were analyzed through peak deconvolution to identify the bonding states constituting the chalcogenide network.

The Ge 3*d* spectrum (Figure 2c) was deconvoluted into five components located at 30.45 eV (Ge─Ge), 32.45 eV (Ge─Se), 33.0 eV (GeO), 34.2 eV (GeO_2_), and 35.4 eV (GeO_x_) [36, 37]. The Ge─Se peak indicates that Ge atoms form heteropolar bonds with Se, while the Ge─Ge peak reveals the presence of homopolar bonds. The GeO, GeO_2_, and GeO_x_ components reflect residual oxide‐related states. The relatively higher binding energy of the Ge─Se peak compared to that of Ge─Ge is consistent with the higher electronegativity of Se, which renders Ge atoms bonded to Se more electron‐deficient.

The detailed fitting parameters of all deconvoluted components are summarized in Table S2. Notably, the Ge─Se component exhibits a FWHM of 1.99 eV, appreciably larger than those of the remaining Ge 3*d* components (1.30–1.39 eV). This width arises from two superimposed contributions. Part of it is common to the entire core level: the Ge 3*d* spin–orbit splitting of 0.585 eV lies close to the energy resolution of the measurement and could not be resolved, so that every Ge 3*d* component represents an unresolved doublet envelope. The remaining broadening, which is specific to the Ge─Se component, instead reflects the distribution of local coordination environments around Ge. Because the film is Se‐deficient, four‐fold coordinated Ge atoms cannot all be fully satisfied by Se neighbors and therefore occupy a range of mixed environments—Ge(Se)_4_, Ge(Se)_3_Ge, Ge(Se)_2_Ge_2_, and so forth—whose slightly different chemical shifts merge into a single broadened envelope. This distribution of local bonding configurations implies a correspondingly dispersed rather than discrete distribution of associated defect‐state energies.

The Se 3*d* spectrum (Figure 2d) shows three pairs of spin–orbit split components corresponding to the Se 3*d_5_/_2_* and 3*d_3_/_2_* doublets. The deconvoluted peaks are assigned to Se─Ge (53.95 eV / 54.85 eV), Se─Sb (54.15 eV / 55.05 eV), and Se─Se (54.25 eV / 55.15 eV) bonding states [38, 39, 40, 41]. This indicates that Se atoms participate in heteropolar bonds with both Ge and Sb, as well as in Se─Se homopolar bonds within the network. The close binding‐energy spacing among these three bonding states reflects the chemically heterogeneous environment surrounding Se atoms, which is expected to play a critical role in trap formation behavior.

The Sb 3*d* spectrum (Figure 2e) was deconvoluted into three components: Sb─Se (530.88 eV / 539.69 eV for 3*d_5_/_2_* and 3*d_3_/_2_*, respectively), Sb─Sb (529.08 eV / 538.34 eV), and SbO_x_ (531.73 eV) [40, 42, 43]. The Sb─Se peak confirms that Sb atoms form heteropolar bonds with Se within the network, consistent with the corresponding Se─Sb component observed in the Se 3*d* spectrum. The Sb─Sb peak indicates the presence of homopolar bonds, while the SbO_x_ component reflects residual oxide‐related states.

Taken together, the XPS results demonstrate that the GeSbSe film is composed of a chalcogenide network in which Ge─Se and Sb─Se heteropolar bonds coexist with Ge─Ge, Se─Se, and Sb─Sb homopolar bonds. The coexistence of these bonding configurations establishes a chemically heterogeneous environment that is expected to govern the local electronic structure and trap formation behavior, providing a structural foundation for the trap‐assisted conduction analysis to be discussed in the following sections.

### 2.2 Electrical Characteristics of GeSbSe Devices

To investigate the electrical characteristics of the GeSbSe‐based device, both direct current (DC) current–voltage (*I–V*) measurements and pulse‐based *I–V* (PIV) measurements were performed. The measurement circuit configurations for the two methods are shown in Figure S1a,b. The DC *I–V* measurements were carried out using a Source Measure Unit (Keithley 2636B), while the PIV measurements were performed using a setup consisting of an Arbitrary Function Generator (Agilent 81150A) and an Oscilloscope (MSOX3024T). For the PIV measurements, a 70 kΩ external series resistor was incorporated into the circuit to limit the peak current and ensure stable operation. The voltage waveforms applied in each measurement method are schematically illustrated in Figure S1c,d. In both DC *I‐V* and PIV measurements, positive (POS) and negative (NEG) write pulses were applied, and the V_th_ was extracted for each state. Throughout this study, blue curves denote the V_th1_ state measured after a POS write operation, while red curves represent the V_th2_ state measured after a NEG write operation.

Figure 3a presents the DC *I–V* characteristics of the GeSbSe device, measured across 10 cells with 4 cycles each to ensure statistical reproducibility. The V_th1_ state after POS write is observed in the range of approximately 0.8–1 V, while the V_th2_ state after NEG write appears in the range of approximately 3.5–4 V, demonstrating a clear separation of V_th_ depending on the polarity of the applied pulse. This polarity‐dependent V_th_ separation directly supports that the GeSbSe device operates beyond simple threshold switching, exhibiting SOM behavior in which the memory state is modulated by the polarity of the applied write pulse.

**FIGURE 3:**
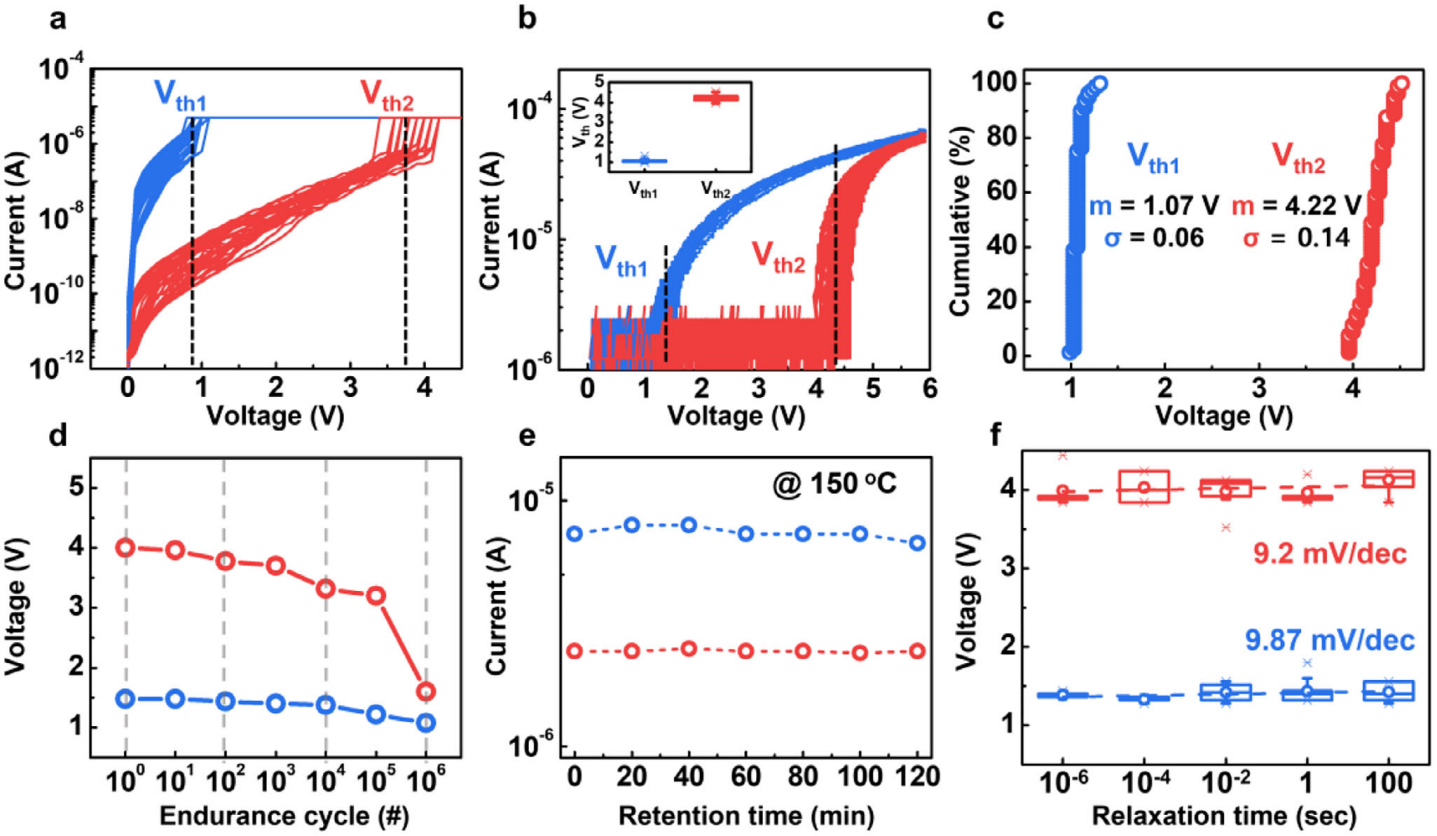
Electrical characteristics and reliability evaluation of the GeSbSe SOM device. (a) DC *I–V* characteristics showing polarity‐dependent threshold switching behavior with distinct V_th1_ and V_th2_ states after POS and NEG write operations, respectively. (b) PIV characteristics of the same device under POS and NEG write conditions. (c) Cumulative probability distributions of V_th1_ and V_th2_ extracted from 80 repeated PIV measurements (10 cells × 8 cycles), with the mean (m) and standard deviation (σ) indicated for each state. (d) Endurance characteristics showing the evolution of V_th1_ and V_th2_ as a function of cycle number. (e) Retention characteristics measured at 150°C, showing the read current levels of the V_th1_ and V_th2_ states as a function of retention time. (f) V_th_ drift characteristics of the V_th1_ and V_th2_ states measured as a function of time after the write pulse.

Figure 3b shows the PIV characteristics of the same device, measured across 10 cells with 8 cycles each. Under PIV conditions, the V_th1_ state is measured at approximately 1 V and the V_th2_ state at approximately 4.2 V, and a clear separation between the two polarity states is consistently observed, in agreement with the DC *I–V* results. The inset in Figure 3b summarizes the mean and standard deviation of V_th1_ and V_th2_ extracted from the PIV measurements, allowing a direct comparison between the two states under identical pulse conditions. Although the same trend of V_th1_ and V_th2_ separation is observed in both DC *I–V* and PIV measurements, the V_th_ values extracted from DC *I–V* are slightly lower than those from PIV. This difference is attributed to the distinct timescales of the two measurement methods. DC sweeps allow more trap‐assisted conduction due to the slower measurement speed, whereas PIV, with its rapid voltage transitions, suppresses the response of slow traps, resulting in a slightly higher apparent V_th._

Figure 3c presents the cumulative probability distributions of V_th1_ and V_th2_ obtained from 80 repeated PIV measurements (10 cells × 8 cycles) on the same device. The V_th1_ state exhibits a mean value of 1.07 V with a standard deviation of 0.06 V, while the V_th2_ state shows a mean value of 4.22 V with a standard deviation of 0.14 V. Both states display very narrow distribution widths, quantitatively confirming that the V_th_ separation between the two polarity states is reproducibly maintained over repeated cycles. These results demonstrate that the GeSbSe device achieves stable SOM operation with low cycle‐to‐cycle variability and a robust V_th_ margin between the two polarity states. To further evaluate the reproducibility of the device characteristics across independent fabrication batches, additional PIV measurements were performed on GeSbSe SOM devices fabricated in two separate batches under identical process conditions, as summarized in Figure S2. For each batch, five cells were characterized over 10 consecutive cycles per cell. Both batches consistently exhibited well‐separated V_th1_ and V_th2_ states, and the corresponding cumulative V_th_ distributions showed comparable mean values and narrow standard deviations. Moreover, all tested cells in both batches exhibited successful polarity‐dependent memory operation. These results demonstrate that the characteristic SOM behavior is reproducibly maintained across multiple cells and independent fabrication batches, supporting the robustness of the device fabrication and memory operation.

To comprehensively evaluate the reliability of the GeSbSe SOM device, endurance, retention, and V_th_ drift characteristics were sequentially measured. The pulse sequences applied for each reliability evaluation are schematically illustrated in Figure S1e–g.

Figure 3d shows the endurance measurement results of the GeSbSe device, tracking the evolution of V_th1_ and V_th2_ over repeated write cycles. As the cycle number increases, both V_th1_ and V_th2_ exhibit a gradual and slight decrease, while the V_th_ separation between the two states is reliably maintained up to approximately 10^5^ cycles. Beyond this point, V_th1_ and V_th2_ converge toward each other near 10^6^ cycles, leading to endurance failure in which the two memory states can no longer be distinguished. These results indicate that the GeSbSe device possesses sufficient cycling robustness against repeated polarity‐pulse stress, while also showing that the gradual accumulation of V_th_ shifts beyond a certain cycle count eventually disrupts the memory state separation.

Figure 3e presents the retention characterization results, which were performed as follows. A write pulse was first applied at room temperature to set the device into either the V_th1_ or V_th2_ state, after which the device was exposed to 150°C for a defined retention time. The device was then returned to room temperature, and a read pulse was applied to evaluate the on/off current levels, thereby assessing the memory state stability under accelerated thermal stress. The measurements confirmed that the read currents for the V_th1_ and V_th2_ states remained clearly distinguishable even after 2 h of exposure at 150°C, with no retention failure observed. This demonstrates that the GeSbSe device sustains robust nonvolatile behavior under accelerated thermal stress conditions.

It is generally known that retention failure in SOM devices is primarily caused by the V_th_ drift phenomenon, in which V_th_ gradually increases over time after the application of the write pulse. As V_th_ shifts toward the read voltage level, located between V_th1_ and V_th2_, over time, or as the separation margin between the two states narrows, read failure eventually occurs. From this perspective, a quantitative analysis of the V_th_ drift characteristics is essential for understanding retention behavior at a more fundamental level.

Figure 3f shows the V_th_ drift results measured on the GeSbSe device. The V_th_ evolution as a function of time after the write pulse was monitored, yielding drift coefficients of 9.87 mV/dec for the V_th1_ state and 9.2 mV/dec for the V_th2_ state. These values represent an excellent level of stability compared with previously reported V_th_ drift values for various SOM devices [32, 44, 45]. Such low drift coefficients quantitatively confirm that the temporal variation of V_th_ is minimal, which can be interpreted as one of the fundamental origins of the excellent retention behavior observed in Figure 3e. In other words, since the GeSbSe device exhibits stable drift characteristics in which V_th_ scarcely increases over time, the memory states can be maintained over extended periods without read failure even under accelerated thermal stress conditions.

Taken together, the results in Figure 3d–f demonstrate that the GeSbSe device operates as a SOM device with reliable performance across all three reliability metrics: endurance, retention, and V_th_ drift. Nevertheless, the gradual V_th_ shifts and eventual endurance failure observed in the endurance measurements, as well as the time‐dependent V_th_ variations associated with retention and V_th_ drift, are all expected to be closely linked to subtle structural and electronic changes occurring within the GeSbSe film. Therefore, in the following section, the physical origins of these reliability behaviors will be investigated by comprehensively employing various physical and electrical analysis techniques to track the evolution of the film state before and after stress from multiple perspectives.

### 2.3 Failure Mechanism Analysis Under Endurance Cycling

#### 2.3.1 Compositional Variation Analysis Under Endurance Cycling

To track the compositional changes within the GeSbSe film under endurance cycling, ToF‐SIMS depth profiling analysis was performed. ToF‐SIMS is a high‐sensitivity analytical technique capable of detecting trace‐level (ppb‐scale) compositional variations within thin films, making it well suited for quantitatively tracking element‐specific compositional fluctuations induced by cycling stress. ToF‐SIMS measurements were carried out on devices subjected to endurance cycling up to 10^0^, 10^2^, 10^4^, and 10^6^ cycles, respectively, and the results are summarized in Figure 4.

**FIGURE 4:**
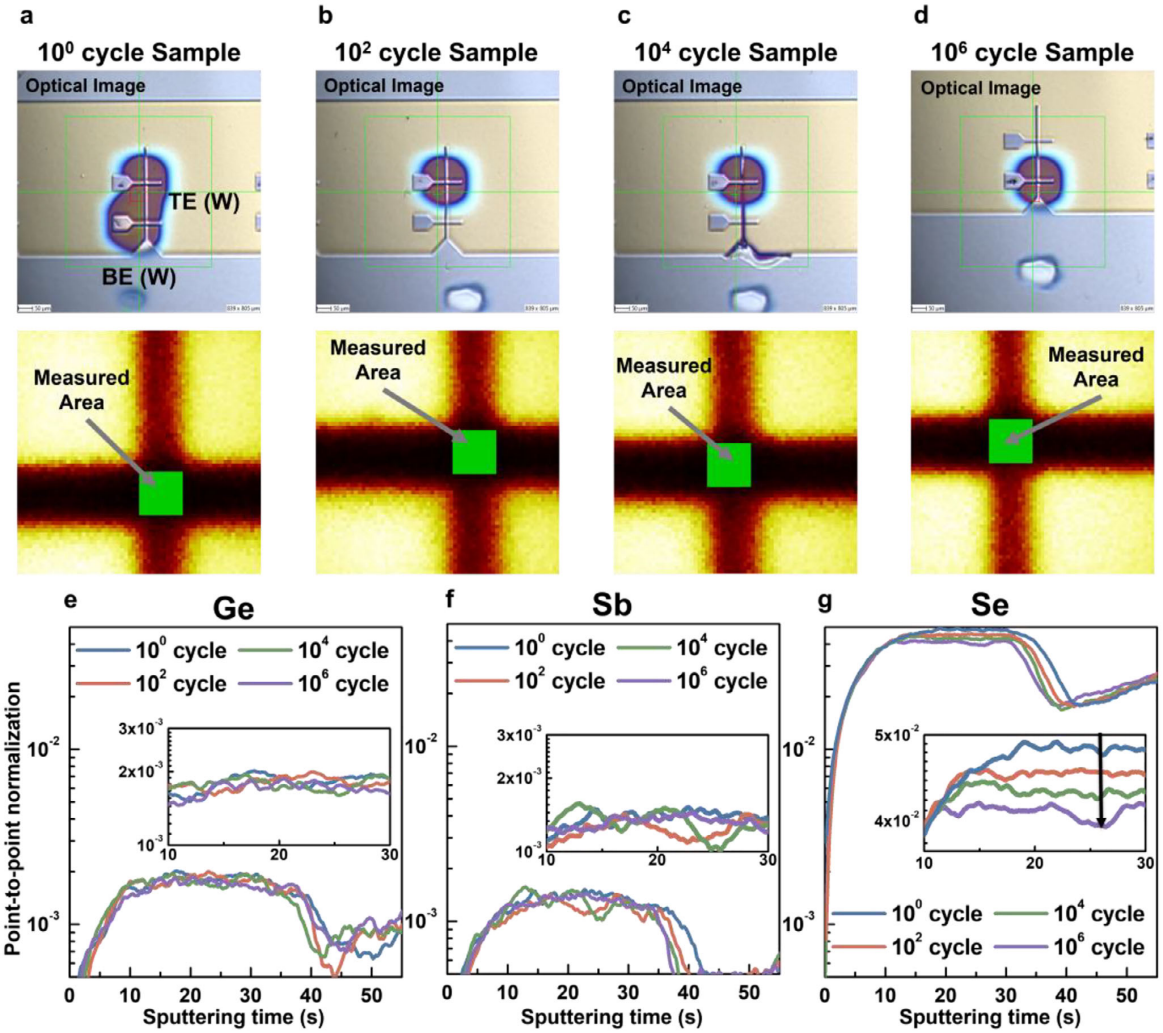
ToF‐SIMS compositional depth profiling analysis of GeSbSe devices subjected to endurance cycling. (a–d) Optical images of the devices at cycle stages of 10^0^, 10^2^, 10^4^, and 10^6^ cycles, respectively, with the corresponding ToF‐SIMS measurement areas indicated by green squares. (e–g) Cycle‐resolved ToF‐SIMS depth profiles of Ge, Sb, and Se, respectively, measured at each cycle stage, with insets showing magnified views of the bulk region to highlight the cycle‐dependent variation in signal intensity.

Figure 4a–d shows the optical images of the devices at each cycle stage (10^0^, 10^2^, 10^4^, and 10^6^ cycles), with the measured area within the active region for ToF‐SIMS analysis indicated by green squares. The devices used for ToF‐SIMS analysis were fabricated with the same 10 × 10 µm^2^ active area and identical stack structure as those used for the electrical measurements. The measured region was carefully positioned at the center of the active area using optical‐microscope alignment and secondary‐electron contrast imaging. Furthermore, measurements were consistently carried out at the same location across all cycle stages, thereby minimizing potential measurement artifacts arising from cycle‐to‐cycle positional deviations or off‐center sampling, and ensuring that only the compositional changes induced by cycling stress itself were reliably extracted.

Figure 4e–g presents the cycle‐resolved depth profiles of Ge, Sb, and Se, respectively. For Ge (Figure 4e) and Sb (Figure 4f), no clear change in signal intensity is observed in the bulk region of the GeSbSe film with increasing cycle number. In contrast, for Se (Figure 4g), the signal intensity in the bulk region exhibits a clear gradual decrease as cycling progresses. This indicates that, while Ge and Sb are essentially preserved within the film during endurance cycling, Se undergoes selective variation under cycling stress.

To more quantitatively assess these element‐specific cycle‐dependent variations, the average signal intensity in the bulk region was extracted for each element and is summarized in Figure S3. Figure S3a–c shows the cycle‐dependent variation of the average signal intensity for Ge, Sb, and Se, respectively. For Ge (Figure S3a), a reduction of approximately 5.9% is obtained between the 1‐cycle and 10^6^‐cycle states, while Sb (Figure S3b) shows a reduction of approximately 4.9%. However, neither element exhibits a consistent monotonic decrease with increasing cycle number, and a slight increase in signal intensity is even observed in certain cycle ranges. In other words, no clear cycling‐induced variation can be identified for Ge and Sb, and even if any variation is present, it remains far less pronounced than the variation observed in Se, as discussed below.

In contrast, for Se (Figure S3c), a reduction of approximately 15.5% is obtained between the 1‐cycle and 10^6^‐cycle states, and the signal intensity decreases monotonically as the cycle number increases. Compared with the negligible‐level variations observed for Ge and Sb, the magnitude and consistent monotonic trend of the Se reduction are clearly distinguishable, directly supporting that Se is genuinely depleted from the bulk region of the GeSbSe film under endurance cycling.

Taken together, the ToF‐SIMS analysis clearly demonstrates that the compositional change induced by endurance cycling in the GeSbSe device does not occur uniformly across all constituent elements, but rather proceeds predominantly through the selective reduction of Se.

This selective variation of Se can be reasonably explained by the intrinsic bonding characteristics of the Se atom. As a group‐VI chalcogen element, Se preferentially forms divalent (two‐fold coordinated) bonds and possesses lone‐pair electrons that do not participate in bonding. Compared with Ge, which favors four‐fold coordination, and Sb, which favors three‐fold coordination, Se atoms are subjected to relatively weaker structural constraints within the network. Consequently, Se atoms occupy sites in which bond switching and local rearrangement can occur more readily under cycling stress. Indeed, prior work on chalcogenide‐based SOM devices has provided direct experimental evidence that Se atoms preferentially migrate toward the BE region under cycling stress, resulting in Se enrichment near the BE [27]. This provides direct experimental evidence that Se is the most preferentially mobile element under cycling stress. Accordingly, the gradual reduction of the Se signal intensity observed in the bulk region of the present GeSbSe system can also be reasonably interpreted as originating from this preferential mobility of Se atoms. This is consistent with the aforementioned prior work reporting Se enrichment near the BE in chalcogenide‐based SOM devices under endurance cycling [27], from which it is inferred that a similar redistribution of Se atoms toward the BE region is likely taking place in the present GeSbSe system as well.

This gradual reduction of Se content is expected to directly influence the bonding environment within the GeSbSe film. As confirmed by the preceding XPS analysis, the GeSbSe film is composed of a network in which Ge─Se and Sb─Se heteropolar bonds coexist with Se─Se, Ge─Ge, and Sb─Sb homopolar bonds. Among these, the Se─Se homopolar bonds are known to form the deepest trap states owing to the strong localization associated with the lone‐pair and anti‐bonding orbitals of Se atoms. The Ge─Se and Sb─Se heteropolar bonds, on the other hand, are reported to provide trap states of intermediate depth, originating from the partial charge localization induced by the electronegativity difference between the constituent atoms. In contrast, the Ge─Ge and Sb─Sb homopolar bonds, which exhibit little asymmetry and a relatively delocalized electron distribution, correspond to comparatively shallow trap states.

Considering this hierarchy of bond‐dependent trap depths, the cycling‐induced reduction of Se content in the GeSbSe layer is likely to preferentially affect the bonding configurations in which Se directly participates, particularly the Se─Se homopolar bonds that contribute to the formation of deep traps. Consequently, as the population of Se‐related bonds responsible for deep trap formation gradually decreases, the overall trap distribution within the film is expected to be reorganized in a direction that lowers the average trap depth, leading to a transition of the trap environment in which the proportion of deep traps decreases while that of shallow traps relatively increases.

This predicted shallow‐direction reorganization is directly corroborated by the electrical measurements presented in Figure S4. As shown in Figure S4a–d, both V_th1_ and V_th2_ decrease progressively with increasing cycle number, consistent with a shallower trap environment requiring a smaller electric field to reach the conduction threshold. Furthermore, Figures S4e and S4f show that the subthreshold off‐current increases monotonically with cycling under both polarity states, reflecting the higher thermal emission probability of carriers from shallower trap states. The concurrent observation of decreasing V_th_ and increasing off‐current thus provides direct electrical evidence corroborating the shallow‐direction trap reorganization predicted from the preceding ToF‐SIMS analysis.

In addition, the change in bonding environment induced by the reduction of Se content is expected to directly influence the memory margin of the SOM device, beyond its impact on the trap distribution. In SOM devices, the V_th_ separation between the two polarity states—namely the memory margin—is generally understood to originate from the asymmetric trap response generated by heteropolar bonds with a relatively large electronegativity difference, such as Ge─Se, under polarity‐dependent pulse operation [46, 47]. Therefore, the gradual reduction of Se atoms induced by cycling is accompanied by a corresponding decrease in the density of key heteropolar bonds, including Ge─Se and Sb─Se, which in turn reduces the bonding resources responsible for generating the polarity‐dependent trap response. Such a change can be directly reflected as a gradual narrowing of the V_th_ separation between the two states with cycling, and provides an interpretation that is qualitatively consistent with the gradual decrease of both V_th1_ and V_th2_ and the progressive shrinkage of the memory margin observed with increasing cycle number in Figure 3d.

It should be emphasized, however, that the above predictions regarding the evolution of trap states and memory margin are qualitative inferences derived from the compositional changes observed in the ToF‐SIMS analysis, and the quantitative variation of trap depth and trap distribution needs to be verified through direct electrical characterization. To this end, the cycle‐dependent variation of trap depth was quantitatively extracted through P–F emission and Arrhenius analyses, and the change in the distribution of deep and shallow traps was subsequently tracked through DLCP analysis, thereby validating the predicted trap‐state evolution from an electrical‐analysis perspective.

#### 2.3.2 Trap‐State Variation Analysis Under Endurance Cycling

The preceding ToF‐SIMS analysis qualitatively predicted that endurance cycling induces a reduction in the Se content within the GeSbSe film, and that such a compositional change can lead to a reorganization of the trap environment in the film. To directly verify this prediction, the cycle‐dependent variation of trap depth was quantitatively extracted. For this purpose, two independent methods—P–F emission model‐based analysis and Arrhenius analysis—were employed in parallel [19, 48]. Although these two methods are based on different physical driving forces, both describe the same trap‐mediated conduction process, so the consistency of the trends extracted from the two analyses can be used to cross‐validate the reliability of the obtained trap depth [49, 50].

This analysis was carried out on GeSbSe devices subjected to endurance cycling up to 10^0^, 10^2^, 10^4^, and 10^6^ cycles, with DC *I–V* measurements performed at four temperatures (25, 35, 45, and 55°C) at each cycle stage. Both *∅_B_* and *E_a_* were extracted by simultaneously applying P–F and Arrhenius fitting to the temperature‐dependent data, and the same procedure was applied independently to both the POS and NEG write states. The P–F model describes a field‐assisted thermal emission process in which the trap barrier is lowered under an applied electric field, expressed as follows in Equation (1) [51, 52].

(1) $$ln\;\left(\frac{J}{E}\right)=\frac{q}{k_{B}T}\;\sqrt{\frac{q}{\pi \varepsilon }}\sqrt{E}-\frac{q}{k_{B}T}\emptyset_{B}+lnC$$

Here, *J* is the current density, *E* is the applied electric field, *q* is the elementary charge, *k_B_* is the Boltzmann constant, *T* is the temperature, *ε* is the permittivity of the film, *∅_B_* is the trap barrier height (i.e., the trap depth), and *C* is a proportionality constant. According to this equation, plotting *ln(J/E)* against *E^1/2^* at a fixed temperature yields a linear relationship, and its y‐intercept is given by Equation (2).

(2) $$y\;intercept=-\emptyset_{B}\frac{q}{k_{B}T}+lnC$$

Therefore, by replotting the y‐intercepts extracted at each temperature against *1/k_B_T*, the *∅_B_* can be quantitatively obtained from the slope of this secondary linear fit. In this study, the *∅_B_* at each cycle stage was extracted through this two‐step fitting procedure.

On the other hand, Arrhenius analysis isolates the influence of the applied electric field and evaluates the thermal energy barrier required for charge carriers trapped in localized states to be released purely through thermal excitation. This analysis is based on the linear dependence of *ln(J/E)* on *1/k_B_T* under a fixed voltage condition, expressed as Equation (3).

(3) $$\ln\left(\frac{J}{E}\right)=\ln\left(C\right)\;-E_{a}\frac{q}{k_{B}T}$$

Here, *E_a_* denotes the activation energy representing the energy barrier that a trapped carrier must overcome to be thermally released into the conduction band. Linear fitting of the *ln(J/E)–1/k_B_T* plot was performed at multiple applied voltage conditions—0.3–1.0 V for the POS write state, as the proximity of V_th1_ to 1 V necessitates strict confinement to the subthreshold regime, and 0.3–1.5 V for the NEG write state where the higher V_th2_ ensures the device remains well within the off‐current regime. The y‐intercept extrapolated to V = 0 was adopted as the representative *E_a_*, allowing direct comparison with *∅_B_* on a consistent physical basis.

Figures S5 and S6 summarize the cycle‐resolved P–F and Arrhenius fitting results under the POS and NEG write states, respectively. The *ln(J/E) vs E^1/2^* plots confirm a well‐satisfied linear relationship at all temperatures, from which *∅_B_* is extracted, and the voltage‐dependent *E_a_* distributions yield the representative zero‐field *E_a_* at each cycle stage.

Figure 5a represents the endurance behavior as a reference, and Figure 5b,c shows the cycle‐dependent variations of *∅_B_* and *E_a_* under both polarity states. Both parameters exhibit a clear monotonic decrease with cycling: under the POS write state, *∅_B_* decreases from 0.332 eV at 10^0^ to 0.080 eV at 10^6^ cycles, and *E_a_* from 0.301 to 0.078 eV. Under the NEG write state, *∅_B_* decreases from 0.510 to 0.128 eV, and *E_a_* from 0.511 to 0.107 eV.

**FIGURE 5:**
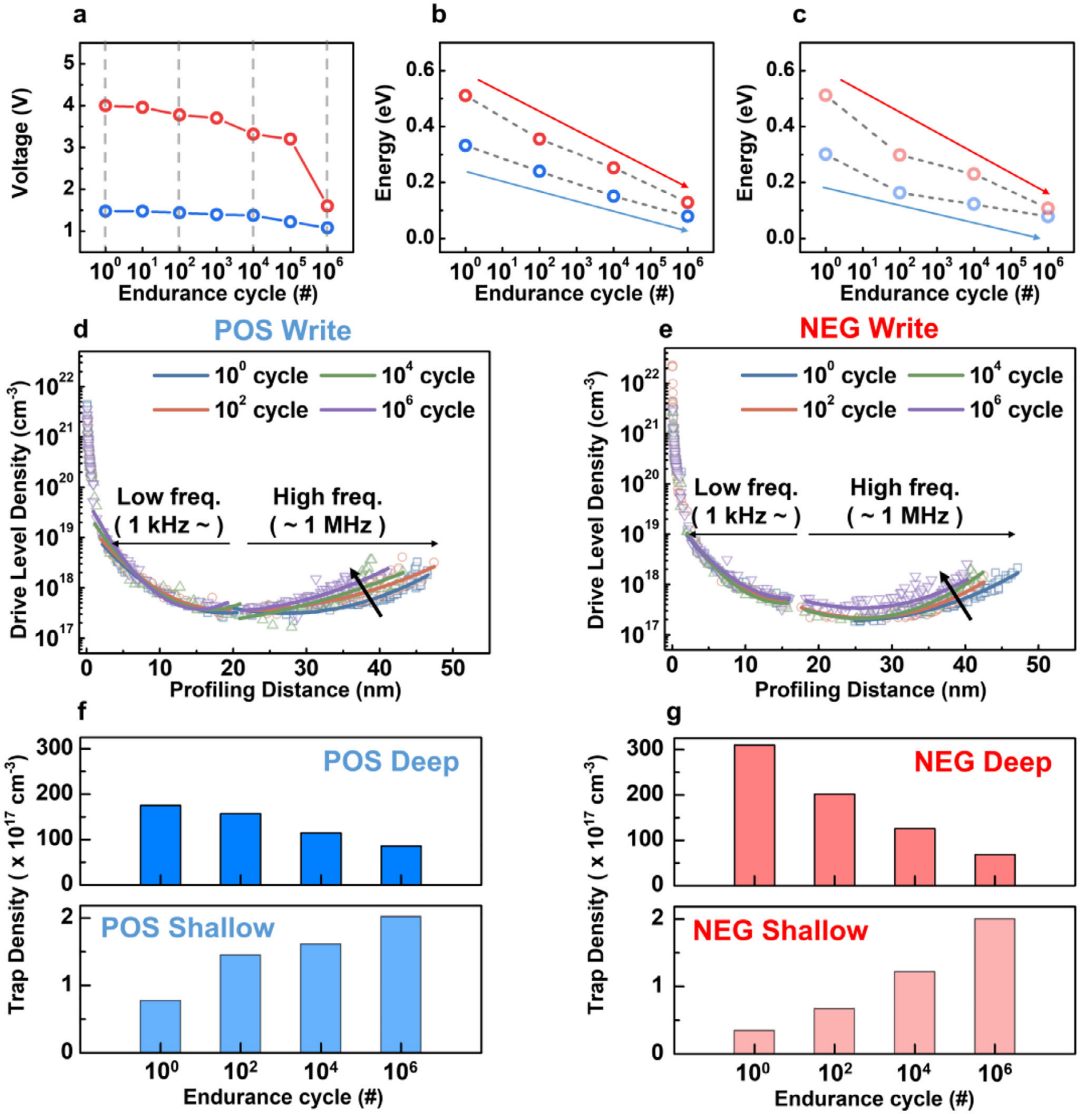
Trap‐state variation analysis of the GeSbSe SOM device under endurance cycling. (a) Cycle‐dependent evolution of V_th1_ and V_th2_, reproduced from Figure 3d as a reference. (b) Cycle‐dependent variation of *∅_B_* extracted from P–F emission analysis for the POS and NEG write states. (c) Cycle‐dependent variation of *E*_a_ extracted from Arrhenius analysis for the POS and NEG write states. (d, e) Frequency‐resolved *N_DL–_d_pf_* profiles measured over a broad frequency range of 1000 Hz–1 MHz at each cycle stage under the POS and NEG write states, respectively. (f, g) Cycle‐dependent variations of deep trap density and shallow trap density extracted by DLCP analysis under the POS and NEG write states, respectively.

Before interpreting these results, the criteria for defining deep and shallow traps need to be clearly established. The *∅_B_* values extracted at the initial state (10^0^ cycles) are adopted as references: 0.510 eV under the NEG write state as the representative deep trap energy, and 0.332 eV under the POS write state as the shallow trap energy. These references are consistently maintained in the subsequent DLCP analysis.

Two key observations can be drawn from Figure 5b,c. First, the convergence of *∅_B_* and *E_a_* to the same monotonic decreasing trend under both polarity states strongly supports that conduction in the GeSbSe device is fundamentally governed by trap‐mediated transport. Second, the monotonic decrease of both parameters directly indicates that the trap environment progressively shifts toward a shallower regime with cycling, with deep traps gradually decreasing and shallow traps correspondingly increasing. Furthermore, the difference in *∅_B_* between the two polarity states also narrows monotonically from 0.178 eV at 10^0^ cycles to 0.048 eV at 10^6^ cycles, and the corresponding *E_a_* difference from 0.21 to 0.029 eV, indicating that the trap asymmetry responsible for separating the two polarity states gradually diminishes, consistent with the V_th_ convergence and endurance failure observed in Figure 5a. These results are in good agreement with the trap‐environment variation predicted from the ToF‐SIMS analysis.

However, while P–F and Arrhenius analyses confirm the shallow‐direction transition of the average trap depth, they cannot independently resolve whether this arises from a decrease in deep traps, an increase in shallow traps, or both. To directly track the variation of the trap distribution by separating it into deep and shallow trap regions, DLCP analysis was performed.

DLCP is a capacitance‐based technique that provides trap distribution information resolved along both the spatial and energetic dimensions by analyzing the capacitance variation under a superposed AC signal of varying amplitude onto a DC bias [53, 54, 55]. In this study, AC amplitude (*V_p‐p_*) was swept from 0.005 to 0.2 V while the AC frequency was varied to separately probe deep and shallow traps according to their response speeds. For each *Vmax* (= DC bias + *V_p‐p_*/2), a *C–V_p‐p_* curve was acquired and fitted with a second‐order polynomial to extract the constant term *C_0_* (measured capacitance) and the first‐order coefficient *C_1_* (voltage sensitivity of capacitance), from which the drive‐level density of localized states (*N_DL_*) is calculated through Equation (4) [56].

(4) $$N_{DL}=-\frac{{C_{0}}^{3}}{2q\varepsilon A^{2}C_{1}}$$

Here, *q* is the elementary charge, *ε* is the permittivity of the film, and *A* is the electrode area. *N_DL_* is not a quantity confined to a single trap level, but rather a volumetric density that reflects the cumulative response of mobile and trapped carriers distributed within a region shallower than the defined profiling distance. Furthermore, the effective profiling distance can be derived from the measured *C_0_* on the basis of the junction capacitance model and is defined as follows in Equation (5) [56].

(5) $$Profiling\;distance\;\;\left(d_{pf}\right)=\frac{\varepsilon A}{C_{0}}$$

That is, regions with larger *C_0_* values probe the shallower part of the film, while those with smaller *C_0_* values probe the deeper part. By simultaneously varying the AC amplitude and frequency in this manner, DLCP can resolve the trap distribution within the film along both the *d_pf_* and the time‐response direction (i.e., frequency, and ultimately energy depth).

To calculate *N_DL_* and the *d_pf_* from the above two equations, the permittivity *ε* (or equivalently, the dielectric constant k) of the film must be quantitatively determined. For this purpose, the DC *I–V* data of the pristine GeSbSe device were analyzed using the P–F emission model to extract the P–F coefficient *β*, and the results are summarized in Figure S7. The *β* value extracted from the linear region of the *ln(J/E)–E^1/2^* plot was 1.387 × 10^−^^5^ eV·m^1/2^·V^−1/2^, and the dielectric constant was subsequently obtained as *k* ≈ 30 using the relation between *β* and *k*,

(6) $$\beta \;=q\;\sqrt{\frac{q}{\pi \varepsilon }}=\sqrt{\frac{q^{3}}{\pi \varepsilon_{0}k}}$$

where *ε_0_* denotes the vacuum permittivity. This value was applied consistently to all DLCP analyses performed in this study.

In addition, the AC frequency and the corresponding trap energy depth are related through the following Equation (7) [57].

(7) $$E=kTln\left(\frac{\omega_{0}}{\omega }\right)$$

Here, *ω* is the measurement angular frequency, and *ω_0_* is the attempt‐to‐escape angular frequency (*τ_0_* = 10^−^^1^ ^2^ s). Physically, only traps whose emission process can be completed within the timescale of the measurement frequency contribute to the capacitance response: low frequencies selectively probe deep traps with slow response speeds, while high frequencies selectively probe shallow traps with fast response speeds. Consequently, *N_DL_* at a given frequency represents a cumulative quantity reflecting all trap states that can respond at that frequency, and by sweeping the frequency over a broad range and converting into the energy domain, the full trap energy distribution can be resolved.

It should be noted that ω_0_ is treated here as a single representative value, whereas in a disordered amorphous network it is expected to vary among trap sites according to the local vibrational environment and capture cross‐section. The influence of this approximation on the present analysis is limited, however, because the energy depends on ω_0_ only logarithmically: an uncertainty of one order of magnitude in ω_0_ corresponds to 0.06 eV, and this shift applies identically to every measurement frequency. The energy axis therefore translates rigidly rather than being distorted, so that the separation between any two points on it, and hence the relative comparison of trap densities on which this work relies, remains unaffected.

Frequency‐resolved *N_DL_* profiles were first measured over a broad range of 1000 Hz–1 MHz to identify the frequency regions corresponding to deep and shallow traps. As shown in Figure 5d,e, the most prominent feature is a gradual increase of *N_DL_* in the high‐frequency region with cycling under both polarity states, consistent with an enhanced shallow trap response as cycling progresses. However, since low‐frequency *N_DL_* reflects a cumulative contribution of both deep and shallow traps, the variation in deep traps cannot be separately identified from these profiles alone. To independently quantify the deep and shallow trap densities and distinguish whether the observed changes arise from new shallow trap generation, transition of deep traps to shallower states, or both, focused measurements over the two identified frequency regions were subsequently performed.

To separately quantify the deep and shallow trap densities, focused measurements were performed at five low‐frequency conditions (1000, 1150, 1380, 1620, and 1900 Hz) for the deep trap region and five high‐frequency conditions (1.0, 1.25, 1.5, 1.75, and 2.0 MHz) for the shallow trap region. For each frequency, a second‐order polynomial fit was applied to the *N_DL–_d_pf_* data, and the *N_DL_* value at a fixed spatial reference point was extracted—*d_pf_* = 0.5 nm for the deep trap region and *d_pf_* = 45 nm for the shallow trap region—to ensure a consistent basis for inter‐frequency comparison. These *N_DL_* values were then converted into the energy domain via Equation 7, constructing *E–N_DL_* curves that separately represent the deep and shallow trap energy regions.

As mentioned earlier, *N_DL_* in DLCP corresponds to the cumulative trap response within the defined probing region, and is therefore expressed as the following integral form of the trap energy distribution *D_t_(E)* in Equation (8) [58].

(8) $$N_{DL}\;\left(E\right)=\int_{0}^{E}D_{t}\left(x\right)dx$$

To extract the local trap density within each energy region, numerical differentiation of *N_DL_* with respect to energy is required. Since the *E–N_DL_* data within each region consist of five discrete points, direct differentiation could introduce numerical instabilities. To address this, the five data points were first interpolated to construct a continuous *N_DL_(E)* curve, and differentiation was subsequently performed to obtain the local trap density *D_t_(E)* as expressed in Equation (9) [58].

(9) $$D_{t}\;\left(E\right)=\frac{dN_{DL}}{dE}*\Delta E$$

This procedure enables *D_t_(E)* to be quantified based on the smoother and more stable rate of change of the continuous interpolated curve, rather than on a simple finite‐difference value calculated directly from the five discrete data points.

Differentiation was carried out at the representative trap energy levels established from the P–F and Arrhenius analyses: *D_t_(E)* near E ≈ 0.510 eV from the low‐frequency data was defined as the cycle‐dependent deep trap density, and *D_t_(E)* near E ≈ 0.332 eV from the high‐frequency data as the cycle‐dependent shallow trap density. This ensures quantitative consistency between the DLCP and P–F/Arrhenius analyses, and by comparing these values cycle by cycle, the variations of the deep and shallow trap densities under cycling stress can be independently quantified and tracked.

The complete DLCP analysis procedures are summarized in Figures S8 and S9 for the POS and NEG write states, respectively. As shown in Figure 5f,g, the deep trap density gradually decreases while the shallow trap density gradually increases with cycling under both polarity states, directly demonstrating that the trap environment within the GeSbSe film transitions from a deep trap‐dominated state to a shallow trap‐dominated state as cycling stress accumulates.

This variation in the trap distribution, driven by cycling‐induced Se atomic migration, provides a consistent trap‐based explanation for the gradual decrease of both V_th1_ and V_th2_ and the progressive narrowing of the memory margin. In summary, Se atomic migration acts as the key additional degradation driving force under endurance cycling, progressively reorganizing the trap environment toward the shallower direction and directly driving the decrease of both V_th1_ and V_th2_. Concurrently, the depletion of Se‐related heteropolar bonds such as Ge─Se weakens the polarity‐dependent asymmetry of the trap response, narrowing the memory margin and ultimately leading to endurance failure near 10^6^ cycles.

### 2.4 Failure Mechanism Analysis Under Retention and Vth Drift

#### 2.4.1 Structural and Chemical Variation Analysis Under Retention and V_th_ Drift

Unlike endurance cycling, where strong electrical stress drives Se atomic migration, retention and V_th_ drift arise from the gradual internal evolution of the film even in the absence of external stress. Since such changes occur very slowly at room temperature, an accelerated evaluation condition of 150°C baking was adopted, which remains sufficiently below the crystallization temperature to preserve the same underlying mechanism. The chemical and structural changes of the GeSbSe film were analyzed as a function of baking time (0, 30, 60, and 90 min) through XPS, Raman spectroscopy, and XRD.

Figure 6a shows the Se 3*d* XPS spectra measured at each baking time, in which the overall peak gradually shifts toward higher binding energies with increasing baking time. This shift reflects a progressive change in the relative contributions of the constituent bonding components: as shown in Figure 6b, the Se─Ge heteropolar bonding peak gradually decreases with baking time, while Figure 6c shows that the Se─Se homopolar bonding peak correspondingly increases. These results directly indicate that the bonding environment of Se atoms is progressively reorganized from heteropolar bonds with Ge toward homopolar Se─Se bonds as baking time elapses. The full deconvolution results of the Ge 3*d* and Sb 3*d* spectra in Figure S10 confirm that all constituent elements evolve consistently in the same direction with baking time.

**FIGURE 6:**
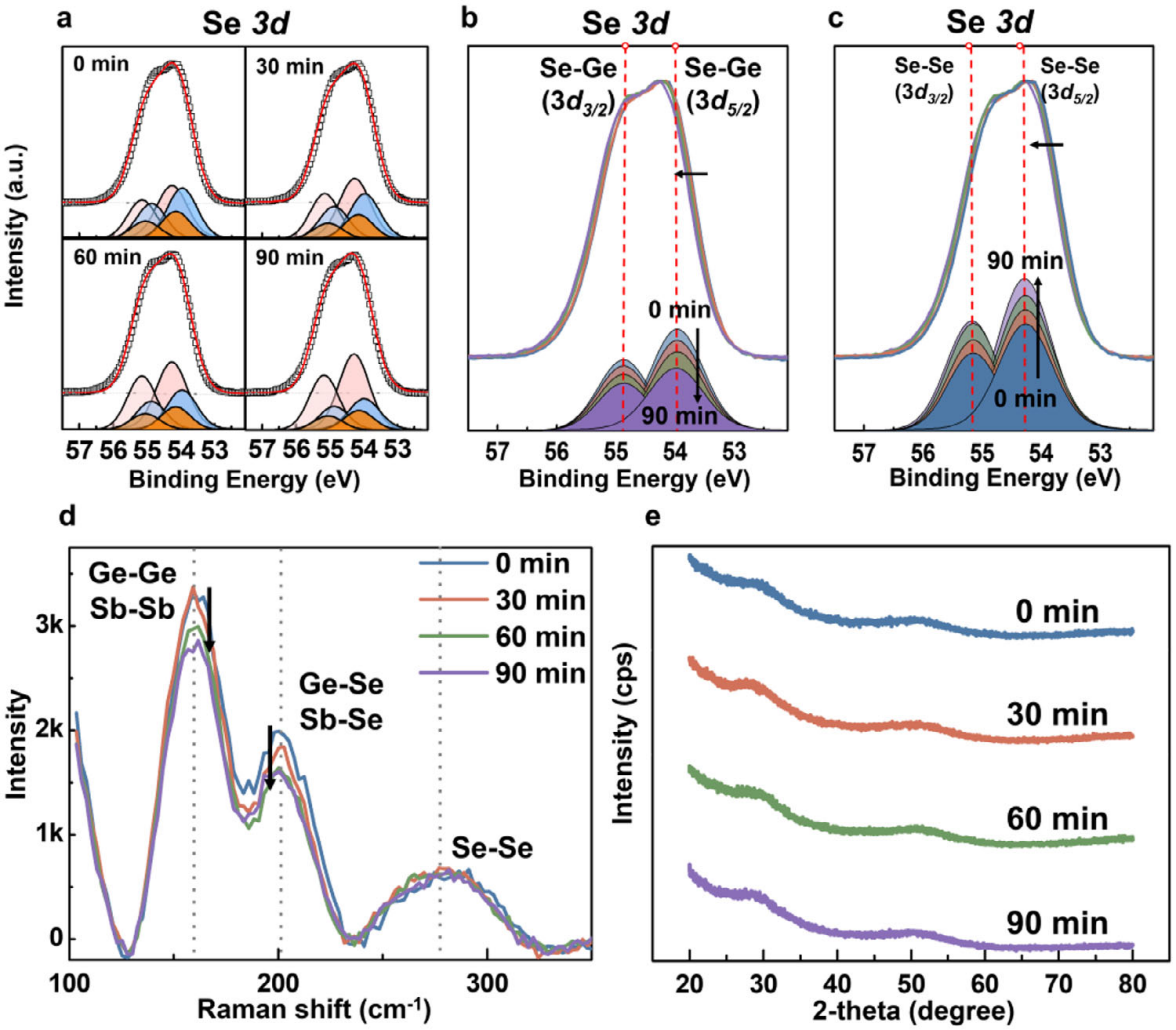
Structural and chemical variation analysis of the GeSbSe film under accelerated evaluation conditions (150°C, 0–90 min). (a) Se 3*d* XPS core‐level spectra with deconvolution results measured at baking times of 0, 30, 60, and 90 min. (b) Baking‐time‐dependent evolution of the Se─Ge bonding peak extracted from the Se 3*d* spectra. (c) Baking‐time‐dependent evolution of the Se─Se bonding peak extracted from the Se 3*d* spectra. (d) Raman spectra of the GeSbSe film measured at each baking time. (e) XRD patterns of the GeSbSe film measured at each baking time.

Figure 6d shows the Raman spectra measured at each baking time, in which the vibrational modes corresponding to Ge─Se and Sb─Se heteropolar bonds and Ge─Ge and Sb─Sb wrong bonds all decrease progressively with baking time. Although no clear variation is observed in the Se─Se vibrational mode—likely due to sensitivity limitations and spectral overlap—the increasing trend of Se─Se bonds is quantitatively supported by the XPS results of Figure 6c. Overall, the Raman analysis confirms that the short‐range bonding environment is progressively reorganized with baking time, with heteropolar and wrong bonds decreasing while Se─Se homopolar bonds become relatively more dominant.

Figure 6e shows the XRD patterns measured at each baking time, in which the GeSbSe film exhibits only a broad amorphous halo without any crystalline diffraction peak at all baking conditions. This confirms that the film stably maintains its amorphous state even under 150°C / 90 min, and that the bonding environment changes observed in the XPS and Raman analyses are confined to short‐range structural reorganization within the amorphous matrix rather than any phase transition.

Taken together, the observations of (i) decreasing Se─Ge heteropolar bonds and increasing Se─Se homopolar bonds, (ii) concurrent decrease of Ge─Ge, Sb─Sb wrong bonds and Ge─Se, Sb─Se heteropolar bonds, and (iii) preservation of the amorphous structure consistently indicate that the variations within the GeSbSe film under 150°C are governed by structural relaxation. Sputtered amorphous chalcogenide films exist in a metastable state with excess wrong bonds and statistical distortions, and upon thermal stimulation below the crystallization temperature, atoms rearrange locally toward a more stable configuration without long‐range migration. In particular, Se—being a group‐VI element with weak network constraints—acts as the most preferentially mobile element, detaching from Se─Ge sites and re‐bonding into Se─Se homopolar configurations, while wrong bonds such as Ge─Ge and Sb─Sb are concurrently redistributed, all confined to short‐range rearrangement within the amorphous matrix.

In contrast to the Se atomic migration observed during endurance cycling—which involves long‐range movement of Se atoms under strong electrical stress, leading to an actual reduction of Se content in the bulk region—the structural relaxation occurring during retention/V_th_ drift corresponds to a short‐range variation in which the overall film composition remains essentially unchanged while only the local bonding environment is reorganized. In both modes, however, these internal variations ultimately affect the trap environment as their consequence.

Applying the bond‐dependent trap‐depth hierarchy established in the endurance analysis, the structural relaxation‐driven bonding changes are expected to directly reorganize the trap environment: the progressive increase of Se─Se bonds raises the deep trap density, while the concurrent decrease of Ge─Se, Sb─Se heteropolar bonds and Ge─Ge, Sb─Sb wrong bonds reduces the intermediate and shallow trap proportions. Consequently, the trap distribution is predicted to shift toward deep trap dominance—the opposite direction from the endurance mode—and this prediction is subsequently verified through P–F, Arrhenius, and DLCP analyses.

The deep‐direction reorganization of the trap environment is expected to constitute the fundamental origin of V_th_ drift: as the trap environment deepens, the electric field required to reach the conduction threshold progressively increases, directly manifesting as a gradual rise of V_th_ with time. Separately, the concurrent decrease of Ge─Se and Sb─Se heteropolar bonds is expected to weaken the asymmetry of the trap response between the two polarity states, resulting in a larger drift magnitude for V_th1_ than for V_th2_ and consequently a gradual narrowing of the memory margin—a scenario supported by the difference in drift coefficients observed in Figure 3f.

Beyond this indirect evidence from the drift coefficient asymmetry, the structural‐analysis‐based prediction of a deep‐direction trap reorganization is more directly supported by the electrical measurements presented in Figure S11. As shown in Figure S11a–d, both V_th1_ and V_th2_ increase progressively with baking time, consistent with a deeper trap environment requiring a larger electric field to reach the trap‐assisted conduction threshold. Furthermore, Figure S11e,f shows that the subthreshold off‐current decreases monotonically with baking time under both polarity states, reflecting the lower thermal emission probability of carriers from progressively deeper trap states. The concurrent observation of rising V_th_ and decreasing off‐current provides mutually consistent and direct electrical evidence corroborating the deep‐direction trap reorganization predicted from the preceding structural analysis.

As in the endurance cycling analysis, P–F and Arrhenius analyses were subsequently performed to quantitatively extract the variation of trap depth, and DLCP analysis was carried out to independently resolve the baking‐time‐dependent evolution of deep and shallow trap densities. In parallel, the variation of V_th1_ and V_th2_ under the same baking conditions was directly measured to confirm how the structural relaxation‐driven trap‐environment reorganization is reflected in the V_th_ drift behavior and memory margin variation.

#### 2.4.2 Trap‐State Variation Analysis Under Retention and V_th_ Drift

Through the preceding physical analysis, it was qualitatively predicted that, under the retention/V_th_ drift mode, the bonding environment within the GeSbSe film progressively evolves through structural relaxation, and the trap environment of the film is consequently reorganized toward the deeper direction. To directly verify this prediction, the variation of the *∅_B_* as a function of baking time was quantitatively extracted. As in the endurance analysis, both the P–F emission model‐based analysis and the Arrhenius analysis were employed. Under the same baking conditions (150°C, 0–90 min), DC *I–V* measurements were performed at four temperatures of 25, 35, 45, and 55°C to obtain the temperature‐dependent conduction behavior, from which *∅_B_* and *E_a_* at each baking time were quantitatively calculated. The same procedure was independently applied to both the POS write and NEG write states in order to track the trap environment variation in each polarity state separately.

Figure 7a shows the variation of V_th1_ and V_th2_ as a function of baking time, in which both states exhibit a clear gradual upward shift with drift coefficients of 124 mV/dec for V_th1_ and 116 mV/dec for V_th2_. The larger drift magnitude of V_th1_ relative to V_th2_ results in a gradual narrowing of the memory margin with increasing baking time.

**FIGURE 7:**
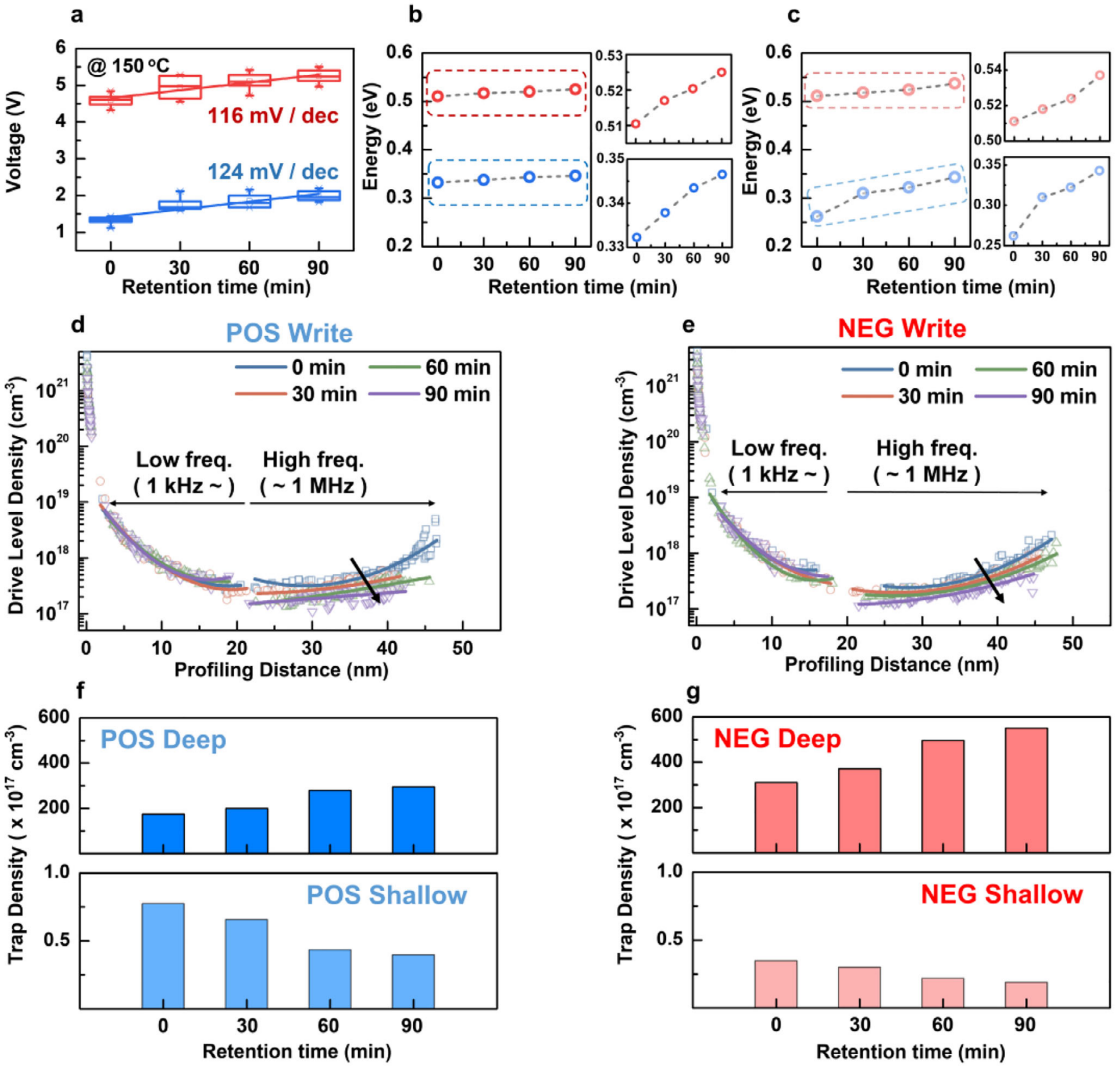
Trap‐state variation analysis of the GeSbSe SOM device under retention and V_th_ drift conditions (150°C, 0–90 min). (a) Baking‐time‐dependent variation of V_th1_ and V_th2_ measured under the accelerated evaluation condition, with drift coefficients indicated for each polarity state. (b) Baking‐time‐dependent variation of *∅_B_* extracted from P–F emission analysis for the POS and NEG write states. (c) Baking‐time‐dependent variation of *E*_a_ extracted from Arrhenius analysis for the POS and NEG write states. (d, e) Frequency‐resolved *N_DL–_d_pf_* profiles measured over a broad frequency range of 1000 Hz–1 MHz at each baking time under the POS and NEG write states, respectively. (f, g) Baking‐time‐dependent variations of deep trap density and shallow trap density extracted by DLCP analysis under the POS and NEG write states, respectively.

As shown in Figure 7b,c, both *∅_B_* and *E_a_* increase monotonically with baking time under both polarity states. Under the POS write state, *∅_B_* increases from 0.332 eV at 0 min to 0.347 eV at 90 min, and *E_a_* from 0.262 to 0.343 eV. Under the NEG write state, *∅_B_* increases from 0.510 to 0.525 eV, and *E_a_* from 0.511 to 0.537 eV. The detailed P–F and Arrhenius fitting results are summarized in Figures S12 and S13 for the POS and NEG write states, respectively.

Two key observations can be drawn from these results. First, *∅_B_* extracted from the P–F fitting and *E_a_* extracted from the Arrhenius fitting exhibit a similar monotonic increasing trend with baking time under both the POS write and NEG write states. Considering that the two analyses are based on different physical driving forces—field‐assisted emission and pure thermal emission, respectively—yet describe the same trap‐mediated conduction process, the convergence of the two results into the same trend strongly supports that the conduction mechanism of the GeSbSe device under the retention mode is fundamentally governed by trap‐mediated behavior, and that the extracted variations of the *∅_B_* represent reliable results.

Second, the fact that both *∅_B_* and *E_a_* monotonically increase with baking time directly indicates that the average trap depth within the GeSbSe film is progressively shifting toward the deeper direction under the retention mode.

While P–F and Arrhenius analyses confirm the deep‐direction transition of the average trap depth, they cannot independently resolve the contributions of deep and shallow traps. DLCP analysis was therefore subsequently employed to directly track the baking‐time‐dependent evolution of each trap density, providing a higher‐resolution verification of the predicted trap‐environment reorganization.

The DLCP measurement principles and analysis methodology described in the endurance cycling analysis were applied in the same manner to the retention/V_th_ drift analysis, with measurements performed under the same baking conditions (150°C, 0–90 min).

As shown in Figure 7d,e, the *N_DL_* values in the high‐frequency region decrease gradually with increasing baking time under both polarity states—in direct contrast to the increasing trend observed during endurance cycling—confirming that the trap environments in the two reliability modes evolve in opposite directions. This is further quantitatively resolved in Figure 7f,g, where the deep trap density gradually increases while the shallow trap density gradually decreases with baking time under both polarity states, directly demonstrating that the trap environment transitions from a shallow trap‐dominated state to a deep trap‐dominated state as retention time elapses. The complete DLCP analysis procedures underlying these results are summarized in Figures S14 and S15 for the POS and NEG write states, respectively.

Synthesizing the results of this series of analyses, all three independent analytical approaches converge to the same conclusion. Physical analysis confirmed that structural relaxation reorganizes the bonding environment, with Se─Se bonds increasing while Ge─Se, Sb─Se, Ge─Ge, and Sb─Sb bonds decrease, predicting a deep‐direction trap reorganization. This was quantitatively verified by the monotonic increase of *∅_B_* and *E_a_* in the P–F and Arrhenius analyses, and directly confirmed at the trap distribution level by the DLCP analysis, in which deep trap density increases and shallow trap density decreases with baking time. The mutual consistency among all three approaches lends strong credibility to the present interpretation.

Furthermore, this deep‐direction reorganization provides a consistent explanation for both the V_th_ drift behavior and the asymmetric drift observed in Figure 7a. The gradual deepening of the trap environment directly drives the progressive rise of V_th1_ and V_th2_ with time, while the concurrent decrease of Ge─Se and Sb─Se heteropolar bonds weakens the asymmetry of the trap response between the two polarity states, resulting in a larger drift magnitude for V_th1_ than for V_th2_ and a gradual narrowing of the memory margin.

Finally, the behaviors observed under the retention/V_th_ drift mode stand in clear contrast to those during endurance cycling: while Se atomic migration under electrical stress reorganizes the trap environment toward shallower states and drives a decrease in V_th_, structural relaxation under thermal stimulation drives the opposite reorganization toward deeper states, leading to a gradual rise in V_th_. Despite evolving in opposite directions, both reliability modes can be consistently explained within the same trap‐based framework, demonstrating that not only the normal operation but also the two distinct major reliability behaviors of the GeSbSe SOM device are governed by a unified mechanistic picture centered on the internal trap environment of the film.

### 2.5 V_th_ Drift Characteristics of Endurance‐Cycled Devices

To further investigate the interplay between endurance cycling and V_th_ drift, V_th_ drift measurements were performed at room temperature on devices subjected to endurance cycling up to 10^0^, 10^2^, 10^4^, and 10^6^ cycles. The measurement scheme is illustrated in Figure 8a, in which a write pulse was first applied to set the device into either the V_th1_ or V_th2_ state following each defined cycle stage, after which the temporal evolution of V_th_ was monitored to extract the drift coefficient. The individual V_th_ drift characteristics at each cycle stage are presented in Figure 8b, with the cycle‐dependent evolution of the drift coefficients and ΔV_th_ drift summarized in Figure 8c.

**FIGURE 8:**
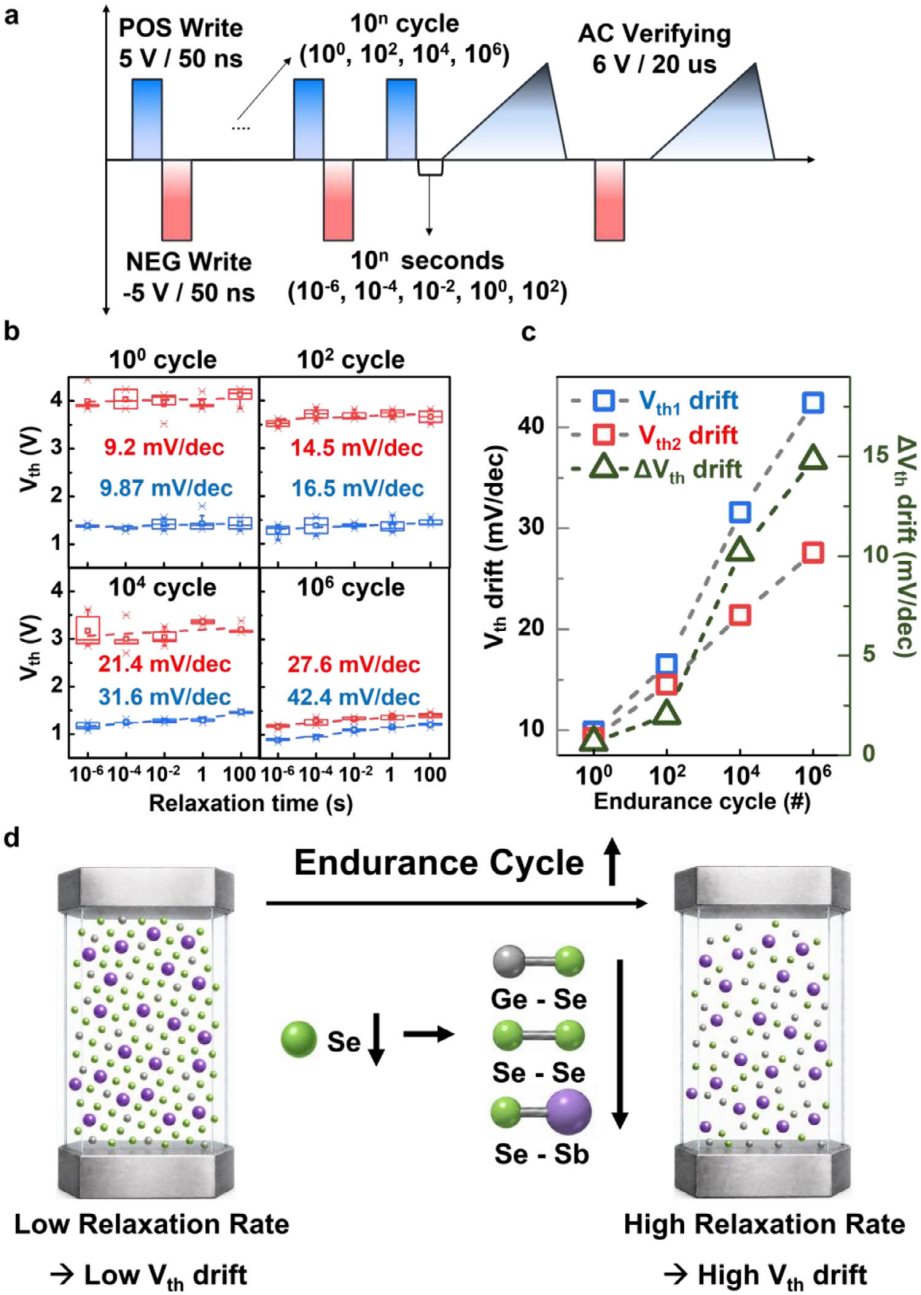
V_th_ drift characteristics of endurance‐cycled GeSbSe SOM devices. (a) Schematic of the measurement scheme for evaluating the interplay between endurance cycling and V_th_ drift. (b) V_th_ drift characteristics of the V_th1_ and V_th2_ states measured at each cycle stage. (c) Cycle‐dependent evolution of the V_th_ drift coefficients and ΔV_th_ drift. (d) Schematic model illustrating the structural origin of the cycling‐accelerated V_th_ drift behavior.

Two clear trends emerge from these results. First, the V_th_ drift coefficients of both V_th1_ and V_th2_ increase progressively with cycle number, from 9.87 and 9.2 mV/dec at 10^0^ cycles to 16.5 and 14.5 mV/dec at 10^2^, 31.6 and 21.4 mV/dec at 10^4^, and 42.4 and 27.6 mV/dec at 10^6^ cycles, respectively. Second, ΔV_th_ drift increases monotonically from 0.67 mV/dec at 10^0^ cycles to 2.0 mV/dec at 10^2^, 10.2 mV/dec at 10^4^, and 14.8 mV/dec at 10^6^ cycles. Since ΔV_th_ drift directly reflects the rate at which the memory margin narrows over time, the progressive increase of ΔV_th_ drift with cycling indicates that the memory margin degrades at an increasingly faster rate as the endurance cycling history accumulates.

Both trends can be consistently interpreted within the unified framework established in the preceding analyses, as schematically illustrated in Figure 8d. Regarding the overall increase of the V_th_ drift coefficient, repeated cycling progressively depletes the total pool of Se‐related bonds, weakening the structural constraints within the amorphous matrix and rendering the remaining bonding configurations increasingly susceptible to thermally driven local rearrangement. Furthermore, as structural relaxation acts upon a progressively smaller bonding reservoir, any given degree of local atomic rearrangement represents a proportionally larger fractional change relative to the remaining bond population, further amplifying its impact on the trap environment per unit time. Through this dual mechanism—network constraint weakening and reduced bonding reservoir—the film transitions from a low to a high relaxation rate state as cycling accumulates, resulting in systematically accelerated V_th_ drift in heavily cycled devices. In other words, endurance cycling progressively lowers the structural resistance of the film against V_th_ drift, so that devices with a larger cycling history exhibit a systematically higher drift rate.

Regarding the progressive increase of ΔV_th_ drift, both endurance cycling and time‐dependent V_th_ drift act to reduce the density of Ge─Se and Sb─Se heteropolar bonds responsible for generating the asymmetric trap response between the two polarity states. In devices that have undergone extensive endurance cycling, the density of these heteropolar bonds is already reduced by cycling‐induced Se migration before any V_th_ drift measurement begins. When structural relaxation subsequently proceeds during V_th_ drift, the same bonding resources are further depleted from an already diminished pool, so that the fractional reduction of heteropolar bonds per unit relaxation becomes progressively larger with cycling history. This progressively weakens the polarity‐dependent asymmetry of the trap response, resulting in a differential drift between V_th1_ and V_th2_ that grows with increasing cycling history. As ΔV_th_ drift increases, the memory margin narrows at a faster rate during the post‐cycling retention period, indicating that the two reliability modes do not act independently but rather reinforce each other in accelerating memory margin degradation.

Taken together, these results demonstrate that the degradation of the GeSbSe SOM device under realistic operating conditions—where endurance cycling and V_th_ drift act simultaneously—is governed by the same trap‐based and bond‐level mechanisms identified in the individual reliability analyses. The progressive depletion of Se‐related bonding resources under cycling stress not only directly induces trap‐environment reorganization during endurance, but also amplifies the susceptibility of the device to subsequent V_th_ drift through a dual mechanism of network constraint weakening and bonding reservoir reduction, establishing a self‐reinforcing degradation pathway that accelerates memory margin loss with accumulated cycling history.

### 2.6 Cross‐Analysis and Unified Trap‐State Evolution Model

Sections 2.3 and 2.4 analyzed endurance and retention degradation as separate reliability modes, and Section 2.5 examined their interaction. To establish that these constitute a single self‐consistent framework, the characteristic parameters obtained from all techniques are placed side by side in Table S3.

Table S3 first summarizes how each degradation mode reorganizes the trap environment. Under endurance cycling, ToF‐SIMS shows that Se is depleted from the film by 15.5%, substantially exceeding the marginal reductions of 5.9% and 4.9% observed for Ge and Sb. Since Se‐related bonds constitute the deep‐trap population within the bond‐dependent trap hierarchy, this preferential loss of Se removes deep traps from the distribution. This is reflected in the monotonic decrease of ∅_B_ and *E_a_* under both write polarities, and confirmed directly at the distribution level by DLCP, in which the deep trap density falls while the shallow trap density rises. The shallowing trap environment in turn lowers V_th_.

Under retention stress, the composition is instead preserved, and the bonding configuration is reorganized. XPS shows that Se─Ge heteropolar bonds progressively convert into Se─Se homopolar bonds, Raman shows that heteropolar and wrong bonds alike decrease, and XRD confirms that the amorphous structure is maintained throughout. Because Se─Se homopolar bonds form the deepest traps, this reorganization adds deep traps to the distribution. Accordingly, ∅_B_ and *E_a_* increase monotonically under both polarities, DLCP resolves an increasing deep trap density with a decreasing shallow trap density, and V_th_ rises.

Placing these two accounts side by side reveals that every observable inverts between the modes, without exception—composition, bonding, trap depth, trap distribution, and device response alike. This systematic inversion establishes that endurance and retention failure are not two manifestations of a common degradation process but two opposite trajectories of trap‐state evolution.

The magnitudes of these changes further reveal that the two trajectories operate on different physical scales. Under endurance cycling, ∅_B_ in the NEG write state falls from 0.510 to 0.128 eV, whereas under retention stress the same parameter rises only from 0.510 to 0.525 eV; the same disparity holds for the POS write state and for *E_a_* under either polarity. The endurance‐driven change is thus more than an order of magnitude larger. This is precisely what the two mechanisms predict. Endurance degradation alters the composition itself, so that the bonds constituting the deep‐trap population are irreversibly removed, and the trap distribution is reconstituted rather than perturbed. Retention degradation proceeds at fixed composition through short‐range rearrangement of existing bonds, redistributing the trap population without changing the inventory of available bonding partners. A compositional mechanism and a configurational one should differ in magnitude by this kind of margin, and the measured values bear this out.

This comparison also bears on the question of whether the observed trap shallowing under endurance cycling can be attributed to field‐induced bond rearrangement rather than to compositional loss. The two are not independent processes: for a Se atom to migrate out of the switching region, its existing bonds must first be broken, and the vacated sites are necessarily reorganized, so bond rearrangement constitutes a step within Se migration rather than a competing mechanism. What can be assessed is which of them governs the magnitude of the observed change. The retention condition provides the one case in which bond rearrangement proceeds in the absence of compositional change, and it therefore establishes a measured scale for the trap‐state change attainable through rearrangement alone—0.015 eV. Accounting for the 0.382 eV change under cycling by rearrangement would require field‐driven reorganization to exceed thermally driven reorganization by more than an order of magnitude, even though the retention condition supplies sustained thermal energy explicitly favorable to bond reorganization. Bond rearrangement is moreover resource‐conserving, since at fixed composition an increase in one bonding configuration comes at the expense of another, whereas Se depletion reduces the total population of Se‐related bonds available to the network. The magnitude of the observed trap shallowing therefore requires the compositional loss of Se as its dominant origin.

Opposing directions, however, do not imply independence. Section 2.5 examined the two modes acting in combination by measuring V_th_ drift on devices subjected to prior endurance cycling, and found that both the drift coefficient and ΔV_th_ drift increase with cycling history. Cycling therefore does not merely shift the trap distribution in the opposite direction to drift; it also accelerates the subsequent drift process itself. This coupling follows directly from the mechanism above: because cycling depletes the reservoir of Se‐related bonds, the remaining network is both less constrained and smaller, so that any given extent of structural relaxation constitutes a proportionally larger perturbation. The two modes thus share a common resource—the Se‐related bonding population—which one consumes and upon which the other acts.

These observations converge on a unified trap‐state evolution model rooted in the as‐deposited structure of the film. As established in Section 2.1, the sputtered GeSbSe layer is Se‐deficient relative to the chemically ordered limit and possesses a mean coordination number well above the rigidity percolation threshold; the network is therefore over‐constrained and inherently contains an excess population of homopolar wrong bonds coexisting with Ge─Se and Sb─Se heteropolar bonds. This heterogeneous inventory defines the bond‐dependent trap hierarchy from which both trajectories originate. Endurance cycling acts upon the inventory by removing its constituents: field‐driven Se migration preferentially eliminates the Se‐related bonds that form deep traps, shifting the distribution toward shallower states. Retention stress acts upon the same inventory by rearranging it: thermal relaxation of the excess wrong bonds drives Se from Se─Ge sites into Se─Se homopolar configurations, shifting the distribution toward deeper states. The decisive distinction is whether the composition is altered—whether the network changes what it is made of, or only how its existing constituents are arranged. This single distinction accounts for the opposite signs of the parameter changes, for their differing magnitudes, and for the accelerating coupling between the two modes, and constitutes the unified bond–trap–reliability framework proposed in this work.

## 3 Conclusions

The reliability failure mechanisms of GeSbSe SOM devices are governed by opposite trap‐state evolution pathways driven by distinct atomic‐scale structural changes, as revealed through a comprehensive combination of physical and electrical analyses.

Under endurance cycling, ToF‐SIMS analysis revealed that repeated electrical stress induces a preferential reduction of Se content in the bulk region of the GeSbSe film, with the Se loss being dominant compared to the other constituent elements. This progressive depletion of Se‐related bonds reorganizes the trap environment toward the shallower direction, as confirmed by the monotonic decrease of *∅_B_* and *E_a_* extracted from P–F and Arrhenius analyses and the cycle‐dependent reduction of deep trap density and increase of shallow trap density independently resolved by DLCP. The gradual shallowing of the trap environment directly drives the decrease of both V_th1_ and V_th2_, while the concurrent loss of heteropolar bonding resources such as Ge─Se and Sb─Se weakens the polarity‐dependent trap response asymmetry, narrowing the memory margin and leading to the eventual endurance failure observed near 10^6^ cycles.

Under retention and V_th_ drift, in contrast, the dominant degradation mechanism shifts from Se atomic migration to short‐range structural relaxation. In the absence of strong external stress, thermal stimulation drives local bond rearrangement within the amorphous matrix, increasing Se─Se homopolar bonds at the expense of heteropolar bonds and wrong bonds. This reorganizes the trap environment in the opposite direction toward deeper traps, as confirmed by the monotonic increase of *∅_B_* and *E_a_* and the baking‐time‐dependent increase of deep trap density and decrease of shallow trap density. The progressive deepening of the trap environment directly drives the time‐dependent rise of V_th_, while the concurrent reduction of Ge─Se and Sb─Se heteropolar bonds accounts for the asymmetric drift between V_th1_ and V_th2_ and the gradual narrowing of the memory margin.

When the two reliability modes act simultaneously, as demonstrated by V_th_ drift measurements performed on endurance‐cycled devices, the cycling‐induced depletion of Se‐related bonding resources amplifies the susceptibility of the film to structural relaxation, systematically accelerating both the overall V_th_ drift rate and the differential drift ΔV_th_ drift with increasing cycling history. This self‐reinforcing degradation pathway establishes that endurance cycling and V_th_ drift are mechanistically coupled, not independent, sharing the same pool of Se‐related bonding resources as a common vulnerability.

Taken together, these findings demonstrate that the normal operation and all major reliability degradation behaviors of the GeSbSe SOM device are governed by a unified trap‐based framework. By revealing that atomic‐scale and bonding‐level changes within the chalcogenide network directly drive the evolution of the trap environment, which in turn determines the trajectory of device‐level electrical degradation, this work establishes a mechanistic foundation for understanding reliability failure in SOM devices. These insights are expected to provide meaningful guidelines for the development of next‐generation SOM technologies with improved endurance and drift stability.

## Author Contributions

B.J.L. conducted the device fabrication, structural analysis, and tested the electrical characteristics. W.H.J. performed the analysis of current conduction mechanism. M.K.Y. and G.H.K. managed the research project and wrote the paper.

## Conflicts of Interest

The authors declare no conflicts of interest.

## Supporting information




**Supporting File**: advs78092‐sup‐0001‐SuppMat.docx.

## Data Availability

The data that support the findings of this study are available from the corresponding author upon reasonable request.
