# Supplementary material for: Opposite Trap‐State Evolution Pathways Govern Endurance and Retention Failure in Amorphous Chalcogenide Memory
Source: Adv Sci (Weinh). 2026 Sep 29:e78092. Online ahead of print. doi: 10.1002/advs.78092 (PMC13624303; doi:10.1002/advs.78092)
Supplement: Supplementary file 1 [file ADVS-9999-e78092-s001.docx]

Supporting Information

**Opposite Trap-State Evolution Pathways Govern Endurance and Retention Failure in Amorphous Chalcogenide Memory**

*Byung Jun Lee^a,1^, Won Hee Jeong^a,1^, Min Kyu Yang^b, *^, and Gun Hwan Kim^a, c,^ **

^a^ Department of System Semiconductor Engineering, Yonsei University, Seoul 03722, Republic of Korea

^b^ Department of Artificial Intelligence Semiconductor Engineering, Sahmyook University, Seoul 01795, Republic of Korea

^c^ Department of Materials Science and Engineering, Yonsei University, Seoul 03722, Republic of Korea

*^*^* Corresponding authors

*^1^*These authors contributed equally to this work

E-mail: Min Kyu Yang ([dbrophd@syu.ac.kr](mailto:dbrophd@syu.ac.kr)), and Gun Hwan Kim ([kgh@yonsei.ac.kr](mailto:kgh@yonsei.ac.kr))

**Experimental**

**1 Device fabrication**

The W/GeSbSe/W SOM devices were fabricated in a two-terminal crossbar-type structure. The defined active area of each unit device is 10 µm × 10 µm. Device fabrication began with the deposition of a 33-nm-thick tungsten (W) film onto a thermally oxidized Si substrate by DC magnetron sputtering. The W BE pattern was defined by photolithography and subsequently transferred by dry etching using inductively coupled plasma reactive-ion etching (ICP-RIE) with an ICP source power of 300 W and a substrate bias power of 50 W. The etching was performed using a mixed gas flow of 4 sccm Ar and 30 sccm Cl₂ at a chamber pressure of 5 mTorr, yielding an etch rate of approximately 1 nm/min. Following etching, residual photoresist was removed by sequential solvent cleaning with acetone, isopropyl alcohol, and deionized water.

The GeSbSe chalcogenide switching layer was deposited by RF co-sputtering using a GeSbSe compound target and an elemental Sb target simultaneously, with applied powers of 38 W and 11 W, respectively, for a deposition time of 400 s. All depositions were carried out under an Ar atmosphere with a working pressure of 3.3 mTorr and a base pressure below 6 × 10⁻⁷ Torr. The co-sputtering approach was adopted to enable precise control of the Sb incorporation ratio within the chalcogenide network while maintaining compositional uniformity across the active layer. The device structure was completed by depositing a 55-nm-thick W TE by DC magnetron sputtering at a power of 140 W for 300 s, with a base pressure of 1 × 10⁻⁶ Torr and a working pressure of 2.1 mTorr under an Ar flow of 15 sccm. The TE pattern was defined by photolithography followed by a standard lift-off process. In all electrical measurements, bias voltage was applied to the W TE while the W BE was grounded.

**2 Material Analysis**

Cross-sectional microstructures of the W/GeSbSe/W SOM devices were characterized using HR-TEM (JEM-ARM200F NEOARM, JEOL) operated at an accelerating voltage of 200 kV with a probe current of 13.9 µA. EDS mapping was performed simultaneously to verify the spatial distribution of constituent elements across the active layer. FFT analysis was applied to the HR-TEM images to confirm the structural state of the chalcogenide film. Cross-sectional TEM specimens were prepared by focused ion beam (FIB) milling using a Crossbeam 350 system (ZEISS).

The elemental composition and areal density of the GeSbSe switching layer were determined by RBS (NEC). A film density of 2.0 × 10¹⁷ atoms/cm² was obtained from the RBS fitting results, and the stoichiometric composition was extracted by simulating the measured scattering profiles.

Chemical bonding states and electronic environments of the constituent elements within the chalcogenide thin films were investigated by XPS (AXIS Supra+, Kratos Analytical) using an Al/Ag dual anode X-ray source with a maximum output of 600 W and 30 mA, and an energy resolution below 0.48 eV at PE10. XPS samples without the W TE were prepared separately, with the GeSbSe layer deposited simultaneously with the device samples under identical conditions. Core-level spectra for Ge 3d, Se 3d, and Sb 3d were acquired and analyzed through peak deconvolution to identify the bonding configurations constituting the chalcogenide network. XPS measurements were also performed on devices subjected to the 150 °C baking conditions (0, 30, 60, and 90 min) used in the retention analysis, in order to track the baking-time-dependent evolution of the bonding environment.

The short-range bonding structure of the GeSbSe film was further examined by Raman spectroscopy (LabRam Soleil, Horiba Jobin Yvon) using a 532 nm excitation laser at a power of 100 mW, covering a spectral range of 30–4000 cm⁻¹. Raman measurements were performed on the same set of baking-condition samples used in the XPS analysis, allowing direct correlation between the vibrational mode evolution and the bonding environment changes observed by XPS.

The crystallographic phase evolution of the GeSbSe film under the accelerated evaluation conditions was monitored by XRD (SmartLab, Rigaku) using Cu Kα-1 radiation generated at a maximum power of 9 kW. Measurements were carried out in the 2θ range from 20° to 80° with a minimum step size of 0.0001° using a vertical θ/θ geometry with a Ge(222) curved crystal monochromator. XRD patterns were acquired for devices exposed to the same 150 °C baking conditions as those used in the XPS and Raman analyses.

Compositional depth profiling under endurance cycling conditions was performed by ToF-SIMS (M6, IONTOF GmbH). Measurements were carried out using a Bi₃⁺ primary ion beam at 30 keV with a current of approximately 0.6 pA for analysis, and a Cs⁺ sputter beam at 1 keV with a current of approximately 30.0 nA for depth profiling, operated in non-interlaced mode with electron flood charge compensation. The analyzer was operated in negative ion mode, and surface potential adjustment was applied to ensure measurement stability. ToF-SIMS depth profiling was performed on devices subjected to endurance cycling of 10⁰, 10², 10⁴, and 10⁶ cycles, respectively, to track the cycle-dependent evolution of elemental distribution within the GeSbSe film.

**3 Electrical characterization**

The threshold switching characteristics of the GeSbSe SOM devices were evaluated using a Source Measure Unit (Keithley 2636B) operated in DC *I–V* sweep mode. PIV measurements were performed using a combined setup comprising an Arbitrary Function Generator (Agilent 81150A) and a Digital Oscilloscope (MSOX3024T). For PIV measurements, a 70 kΩ external series resistor was incorporated into the circuit to limit the peak current and ensure stable switching operation. In all measurements, voltage bias was applied to the W TE while the W BE was grounded.

To investigate the temperature dependence of the subthreshold conduction mechanism, DC *I–V* measurements were carried out on a programmable hot stage with precise temperature control at four temperatures of 25, 35, 45, and 55 °C. From the temperature-dependent *I–V* data, P–F emission analysis was performed by plotting *ln(J/E)* versus *E¹/²* at each temperature condition, and the *∅_B_* was extracted through a two-step fitting procedure in which the y-intercepts obtained from the linear fits were subsequently plotted against *1/k_B_T*, with the slope of the secondary linear fit yielding the effective *∅_B_*. Arrhenius analysis was additionally performed by plotting *ln(J/E)* against *1/k_B_T* under fixed voltage conditions, from which the *E_a_* was extracted by linear fitting. To ensure a reliable and field-independent estimate of *E_a_,* linear fitting was performed at multiple applied voltage conditions, and the resulting voltage-dependent *E_a_* values were linearly fitted and extrapolated to V = 0 to obtain the representative zero-field *E_a_* at each measurement condition. The same P–F and Arrhenius analysis procedures were applied independently to both the POS write and NEG write states, and were carried out at each cycle stage for the endurance analysis and at each baking time for the retention/V_th_ drift analysis.

To investigate the spatial and energetic distribution of trap states within the GeSbSe film, DLCP measurements were performed using a Keysight E4980A Precision LCR Meter. The *V_p-p_* was swept from 0.005 V to 0.2 V with a step size of 0.015 V, and the AC frequency was varied over a range from 1000 Hz to 2 MHz. For each *V_max_* condition, a *C–V_p-p_* curve was acquired, and the constant term *C₀* and the first-order coefficient *C₁* were extracted by second-order polynomial fitting of each curve. These parameters were used to calculate the *N_DL_* and the *d_pf_* according to the equations described in the main text. The dielectric constant of the GeSbSe film used in the DLCP calculations was determined from the P–F coefficient *β* extracted from the DC *I–V* data of the pristine device. DLCP measurements were performed independently under both POS write and NEG write states, and were carried out at each cycle stage for the endurance analysis and at each baking time for the retention/V_th_ drift analysis.

Endurance characteristics were evaluated by applying repeated POS and NEG write pulses, and the threshold voltages V_th1_ and V_th2_ were monitored as a function of cycle number up to 10⁶ cycles. Retention characteristics were assessed by first programming the device into either the V_th1_ or V_th2_ state at room temperature, followed by exposure to 150 °C for up to 2 hours, after which the memory state stability was confirmed by evaluating the on/off current separation using a read pulse at room temperature. V_th_ drift characteristics were evaluated by monitoring the temporal evolution of V_th1_ and V_th2_ following write pulse application, and the drift coefficient was extracted from the linear slope of the V_th_ versus log(time) plot. To investigate the interplay between endurance cycling and V_th_ drift, V_th_ drift measurements were additionally performed at room temperature on devices that had been subjected to endurance cycling of 10⁰, 10², 10⁴, and 10⁶ cycles, respectively.

**Figure S1.** **Measurement circuit configurations and pulse schemes used in this study.** (a) Schematic of the DC measurement circuit using a Source Measure Unit (Keithley 2636B). (b) Schematic of the pulse measurement circuit comprising an Arbitrary Function Generator (Agilent 81150A), an Oscilloscope (MSOX3024T), and a 70 kΩ external series resistor. (c) Voltage pulse scheme applied for DC *I–V* measurements. (d) Voltage pulse scheme applied for PIV measurements. (e) Pulse scheme applied for endurance measurements. (f) Pulse scheme applied for retention measurements. (g) Pulse scheme applied for V_th_ drift measurements.

**Figure S2. Batch-to-batch reproducibility of the SOM devices.** (a,b) PIV characteristics of SOM cells fabricated in two independent batches under identical process conditions (Batch 1 and Batch 2, respectively). For each batch, five cells were measured over 10 consecutive cycles per cell. (c,d) Corresponding cumulative distributions of the threshold voltages (V_th1_ and V_th2_) extracted from the PIV measurements of Batch 1 and Batch 2, respectively. The mean (m) and standard deviation (σ) of each V_th_ distribution are indicated. The well-separated V_th_ distributions with comparable statistical characteristics across the two independently fabricated batches demonstrate the batch-to-batch reproducibility of the SOM devices.

**Figure S3.** Cycle-dependent variation of the average ToF-SIMS signal intensity in the bulk region for (a) Ge, (b) Sb, and (c) Se, extracted from the depth profiles shown in Figure 4.

**Figure S4.** **Electrical characteristics of the GeSbSe SOM device measured at each endurance cycle stage.** (a–d) PIV characteristics measured at cycle stages of 10⁰, 10², 10⁴, and 10⁶ cycles, respectively, showing the progressive decrease of both Vth1 and Vth2 with increasing cycle number. (e, f) Subthreshold off-current characteristics measured under the POS write and NEG write states, respectively, showing the monotonic increase of leakage current with cycling under both polarity states.

**Figure S5.** **Cycle-resolved P–F emission and Arrhenius fitting results under the POS write state for devices subjected to endurance cycling at 10⁰, 10², 10⁴, and 10⁶ cycles.** (a, d, g, j) ln(*J*/*E*) vs *E*¹/² plots measured at four temperatures (25, 35, 45, and 55 °C) at each cycle stage, used for P–F emission analysis. (b, e, h, k) y-intercept vs *1/k_B_T* plots extracted from (a, d, g, j), from which the *∅_B_* is obtained. (c, f, i, l) Voltage-dependent *E*_a_ extracted from Arrhenius fitting at each cycle stage.

**Figure S6.** **Cycle-resolved P–F emission and Arrhenius fitting results under the NEG write state for devices subjected to endurance cycling at 10⁰, 10², 10⁴, and 10⁶ cycles.** (a, d, g, j) ln(*J*/*E*) vs *E*¹/² plots measured at four temperatures (25, 35, 45, and 55 °C) at each cycle stage, used for P–F emission analysis. (b, e, h, k) y-intercept vs *1/k_B_T* plots extracted from (a, d, g, j), from which the *∅_B_* is obtained. (c, f, i, l) Voltage-dependent *E*_a_ extracted from Arrhenius fitting at each cycle stage.

**Figure S7. Extraction of the P–F coefficient *β* and dielectric constant *k* of the pristine GeSbSe device.** (a) ln(*J*/*E*) vs *E*¹/² plot of the pristine GeSbSe device, from which the P–F coefficient *β* is extracted from the linear region. (b) Relationship between *β* and the dielectric constant *k* derived using Equation 6, yielding *k* ≈ 30.

**Figure S8.** **Cycle-resolved DLCP measurement and analysis results under the POS write state for devices subjected to endurance cycling at 10⁰, 10², 10⁴, and 10⁶ cycles.** (a, e, i, m) *N_DL_–d_pf_* profiles measured at five low-frequency conditions (1000, 1150, 1380, 1620, and 1900 Hz) for deep trap probing at each cycle stage. (b, f, j, n) Corresponding *N_DL_–E* curves converted into the energy domain from the low-frequency measurements. (c, g, k, o) *N_DL_–d_pf_* profiles measured at five high-frequency conditions (1.0, 1.25, 1.5, 1.75, and 2.0 MHz) for shallow trap probing at each cycle stage. (d, h, l, p) Corresponding *NDL–E* curves converted into the energy domain from the high-frequency measurements.

**Figure S9.** **Cycle-resolved DLCP measurement and analysis results under the NEG write state for devices subjected to endurance cycling at 10⁰, 10², 10⁴, and 10⁶ cycles.** (a, e, i, m) *N_DL_–d_pf_* profiles measured at five low-frequency conditions (1000, 1150, 1380, 1620, and 1900 Hz) for deep trap probing at each cycle stage. (b, f, j, n) Corresponding *N_DL_–E* curves converted into the energy domain from the low-frequency measurements. (c, g, k, o) *N_DL_–d_pf_* profiles measured at five high-frequency conditions (1.0, 1.25, 1.5, 1.75, and 2.0 MHz) for shallow trap probing at each cycle stage. (d, h, l, p) Corresponding *N_DL_–E* curves converted into the energy domain from the high-frequency measurements.

**Figure S10.** **Baking-time-resolved XPS core-level spectra with deconvolution results for the GeSbSe film exposed to 150 °C for 0, 30, 60, and 90 min.** (a, d, g, j) Ge 3*d* core-level spectra with deconvoluted peak components at each baking time. (b, e, h, k) Se 3*d* core-level spectra with deconvoluted peak components at each baking time. (c, f, i, l) Sb 3*d* core-level spectra with deconvoluted peak components at each baking time.

**Figure S11. Electrical characteristics of the GeSbSe SOM device measured under accelerated evaluation conditions (150 °C, 0–90 min).** (a–d) PIV characteristics measured after baking durations of 0, 30, 60, and 90 min, respectively, showing the progressive increase of both V_th1_ and V_th2_ with baking time. (e, f) Subthreshold off-current characteristics measured under the POS write and NEG write states, respectively, showing the monotonic decrease of leakage current with baking time under both polarity states.

**Figure S12.** **Baking-time-resolved P–F emission and Arrhenius fitting results under the POS write state for devices subjected to the accelerated evaluation condition (150 °C) for 0, 30, 60, and 90 min.** (a, d, g, j) ln(*J*/*E*) vs *E*¹/² plots measured at four temperatures (25, 35, 45, and 55 °C) at each baking time, used for P–F emission analysis. (b, e, h, k) y-intercept vs *1/k_B_T* plots extracted from (a, d, g, j), from which the *∅_B_* is obtained. (c, f, i, l) Voltage-dependent *E*_a_ extracted from Arrhenius fitting at each baking time.

**Figure S13.** **Baking-time-resolved P–F emission and Arrhenius fitting results under the NEG write state for devices subjected to the accelerated evaluation condition (150 °C) for 0, 30, 60, and 90 min.** (a, d, g, j) ln(*J*/*E*) vs *E*¹/² plots measured at four temperatures (25, 35, 45, and 55 °C) at each baking time, used for P–F emission analysis. (b, e, h, k) y-intercept vs *1/k_B_T* plots extracted from (a, d, g, j), from which the *∅_B_* is obtained. (c, f, i, l) Voltage-dependent *E*_a_ extracted from Arrhenius fitting at each baking time.

**Figure S14.** **Baking-time-resolved DLCP measurement and analysis results under the POS write state for devices subjected to the accelerated evaluation condition (150 °C) for 0, 30, 60, and 90 min.** (a, e, i, m) *N_DL_–d_pf_* profiles measured at five low-frequency conditions (1000, 1150, 1380, 1620, and 1900 Hz) for deep trap probing at each baking time. (b, f, j, n) Corresponding *N_DL_–E* curves converted into the energy domain from the low-frequency measurements. (c, g, k, o) *N_DL_–d_pf_* profiles measured at five high-frequency conditions (1.0, 1.25, 1.5, 1.75, and 2.0 MHz) for shallow trap probing at each baking time. (d, h, l, p) Corresponding *N_DL_–E* curves converted into the energy domain from the high-frequency measurements.

**Figure S15.** **Baking-time-resolved DLCP measurement and analysis results under the NEG write state for devices subjected to the accelerated evaluation condition (150 °C) for 0, 30, 60, and 90 min.** (a, e, i, m) *N_DL_–d_pf_* profiles measured at five low-frequency conditions (1000, 1150, 1380, 1620, and 1900 Hz) for deep trap probing at each baking time. (b, f, j, n) Corresponding *N_DL_–E* curves converted into the energy domain from the low-frequency measurements. (c, g, k, o) *N_DL_–d_pf_* profiles measured at five high-frequency conditions (1.0, 1.25, 1.5, 1.75, and 2.0 MHz) for shallow trap probing at each baking time. (d, h, l, p) Corresponding *N_DL_–E* curves converted into the energy domain from the high-frequency measurements.

| **Table S1.** Quantitative composition of the GeSbSe switching layer determined by RBS and EDS | | | |
| --- | --- | --- | --- |
| **Technique** | **Ge**  **(at.%)** | **Sb**  **(at.%)** | **Se**  **(at.%)** |
| **RBS** | 33.0 | 17.0 | 50.0 |
| **EDS**  **(STEM)** | 38.1 | 14.7 | 47.2 |

| **Table S2.** Peak fitting parameters of the Ge 3d, Se 3d and Sb 3d core-level XPS spectra of the as-deposited GeSbSe film | | | | |
| --- | --- | --- | --- | --- |
| **Core level** | **Component** | **BE (eV)** | **FWHM (eV)** | **Relative area (%)** |
| Ge 3*d* | Ge-Ge | 30.45 | 1.39 | 31.30 |
|  | Ge-Se | 32.45 | 1.99 | 27.05 |
|  | GeO | 33.0 | 1.39 | 25.57 |
|  | GeO_2_ | 34.2 | 1.30 | 14.47 |
|  | GeO_x_ | 35.4 | 1.30 | 1.61 |
| Se 3*d* | Se-Ge | 53.95 / 54.85 | 1.05 | 38.54 |
|  | Se-Sb | 54.15 / 55.05 | 1.00 | 19.25 |
|  | Se-Se | 54.25 / 55.15 | 1.00 | 42.21 |
| Sb 3*d* | Sb-Sb | 529.08 / 538.34 | 1.54 | 72.03 |
|  | Sb-Se | 530.88 / 539.69 | 1.41 | 12.15 |
|  | SbO_x_ | 531.73 | 1.57 | 15.82 |

| **Table S3**. Cross-technique comparison of the two reliability degradation modes | | |
| --- | --- | --- |
| **Technique** | **Endurance**  **(10^0^ 🡪 10^6^ cycles)** | **Retention**  **(150 ^o^C 0 🡪 90 min)** |
| ToF-SIMS | Se -15.5%  Ge -5.9 %  Sb -4.9% | -- |
| XPS | -- | Se-Ge decrease  Se-Se increase |
| Raman | -- | Ge-Se, Sb-Se, Ge-Ge,  Sb-Sb decrease |
| XRD | -- | Amorphous structure preserved |
| P-F (*∅_B_*) | 0.510 🡪 0.128 eV (NEG)  0.332 🡪 0.080 eV (POS) | 0.510 🡪 0.525 eV (NEG)  0.332 🡪 0.347 eV (POS) |
| Arrhenius (*E_a_*) | 0.511 🡪 0.107 eV (NEG)  0.301 🡪 0.078 eV (POS) | 0.511 🡪 0.537 eV (NEG)  0.262 🡪 0.343 eV (POS) |
| DLCP | Deep trap ↓  Shallow trap ↑ | Deep trap ↑  Shallow trap ↓ |
| I-V | V_th_ decrease | V_th_ increase |

ToF-SIMS was applied to the endurance mode, in which compositional change governs the degradation, whereas XPS, Raman, and XRD were applied to the retention mode, in which the composition is preserved and only the bonding configuration is reorganized.
